# Unit-cell-thick zeolitic imidazolate framework films for membrane application

**DOI:** 10.1038/s41563-023-01669-z

**Published:** 2023-09-21

**Authors:** Qi Liu, Yurun Miao, Luis Francisco Villalobos, Shaoxian Li, Heng-Yu Chi, Cailing Chen, Mohammad Tohidi Vahdat, Shuqing Song, Deepu J. Babu, Jian Hao, Yu Han, Michael Tsapatsis, Kumar Varoon Agrawal

**Affiliations:** 1 Laboratory of Advanced Separations, École Polytechnique Fédérale de Lausanne (EPFL), Sion, Switzerland; 2 College of Chemistry, Chemical Engineering and Materials Science, Soochow University, Suzhou, China; 3 Department of Chemical and Biomolecular Engineering & Institute for NanoBioTechnology, Johns Hopkins University, Baltimore, MD USA; 4 Advanced Membranes and Porous Materials Center, Physical Sciences and Engineering Division, King Abdullah University of Science and Technology, Thuwal, Saudi Arabia; 5 Applied Physics Laboratory, Johns Hopkins University, Laurel, MD USA; 6 Present Address: Mork Family Department of Chemical Engineering and Materials Science, University of Southern California, Los Angeles, California USA; 7 Present Address: Materials Science and Metallurgical Engineering, Indian Institute of Technology, Hyderabad, India

**Keywords:** Two-dimensional materials, Metal-organic frameworks, Chemical engineering, Nanopores

## Abstract

Zeolitic imidazolate frameworks (ZIFs) are a subset of metal–organic frameworks with more than 200 characterized crystalline and amorphous networks made of divalent transition metal centres (for example, Zn^2+^ and Co^2+^) linked by imidazolate linkers. ZIF thin films have been intensively pursued, motivated by the desire to prepare membranes for selective gas and liquid separations. To achieve membranes with high throughput, as in ångström-scale biological channels with nanometre-scale path lengths, ZIF films with the minimum possible thickness—down to just one unit cell—are highly desired. However, the state-of-the-art methods yield membranes where ZIF films have thickness exceeding 50 nm. Here we report a crystallization method from ultradilute precursor mixtures, which exploits registry with the underlying crystalline substrate, yielding (within minutes) crystalline ZIF films with thickness down to that of a single structural building unit (2 nm). The film crystallized on graphene has a rigid aperture made of a six-membered zinc imidazolate coordination ring, enabling high-permselective H_2_ separation performance. The method reported here will probably accelerate the development of two-dimensional metal–organic framework films for efficient membrane separation.

## Abstract (web summary)

Unit-cell-thick films of metal–organic frameworks with ordered porosity would be attractive for membrane applications as these thin systems combine large molecular flux with high selectivity. Here crystalline ZIF films are grown on a crystalline substrate with high H_2_/N_2_ gas separation performance.

## Main

Zeolitic imidazolate frameworks (ZIFs)^1,2^ are a class of metal–organic frameworks (MOFs) that hold promise for applications in molecular separations^3–8^, patterning^9,10^ and sensing^11^. Their chemical and physical properties have been widely explored as a function of framework flexibility^12–15^ as well as structural defects^16,17^. The realization of two-dimensional (2D) ZIF films with thickness down to that afforded by a single structural building unit is highly desired to make ZIF analogues to graphene and related 2D materials with an added advantage: the intrinsic nanoporosity of ZIF can be used to separate molecules and maximize the permselective flux^18^. However, the realization of 2D crystalline and ultrathin amorphous ZIF films has remained elusive. Although layered ZIFs such as ZIF-L (ref. ^19^), Zn_2_(bim)_4_ (ref. ^20^) and analogues^21^ have been reported, individual ZIF layers in these materials have a small aspect ratio, which prevents the realization of continuous 2D ZIF films with structural uniformity over a macroscopic (for example, wafer) length scale. State-of-the-art ZIF deposition methods yield polycrystalline films with thickness larger than 50 nm (refs. ^22–25^). This is mainly due to difficulty in achieving in-plane film growth without film thickening.

Considerable knowledge exists on ZIF/MOF crystal nucleation and growth in solution^26–31^. Based on data from synchrotron X-ray scattering, density functional theory (DFT) and molecular dynamics simulations, as well as other techniques, it is generally accepted that ZIF formation involves a sequence of events starting from the formation of small (~1 nm) metastable prenucleation clusters, which evolve through aggregation followed by intra-aggregate ZIF nucleation and growth. Recent studies on surface-directed MOF growth^32–40^ indicate that the diffusion of MOF precursors in the vicinity of the 2D material and MOF–2D material interactions are key to regulate the crystallinity of the MOF film and the ability to maintain in-plane/horizontal growth (desired for ultrathin films) versus out-of-plane/vertical (undesired) growth.

Here we report macroscopically uniform 2D ZIF films with exquisite nanometre-scale control over the film thickness by suppressing the out-of-plane growth by using an ultradilute growth solution. The ultralow precursor concentration restricts homogeneous nucleation in the solution and facilitates the growth of nanometre-thick films over an immersed substrate with deposition timescales of a few minutes. The film crystallinity is determined by the interaction of molecular precursors with the substrate ranging from substrate-registry-determined order to amorphous films in the absence of any crystallographic registry. The film thickness could be controlled with a resolution of a single layer by controlling the deposition time and number of coatings.

## Synthesis and characterization of 2DZIF film

**Fig. 1:**
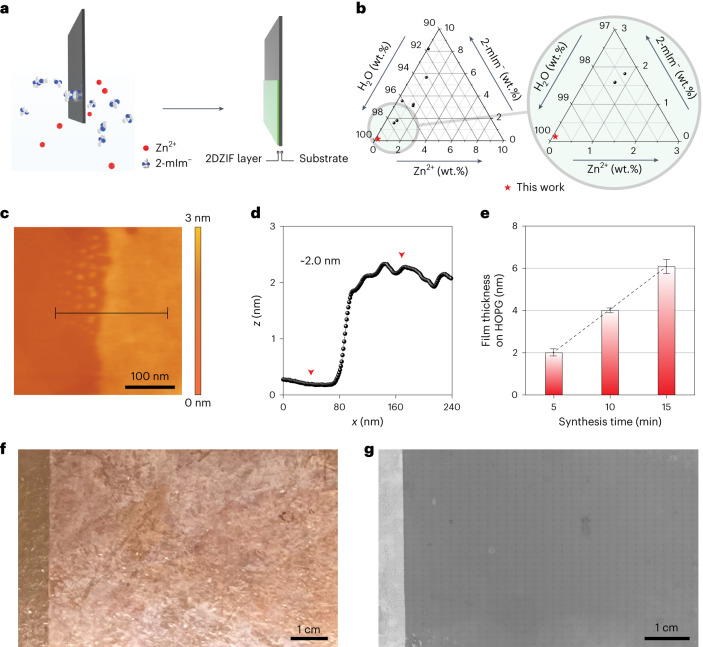
Synthesis of ZIF films from ultradilute solutions. **a**, Schematic of ZIF film synthesis. **b**, Composition diagram comparing the precursor solution composition used in this study with those reported in the literature (Supplementary Table 1). **c**,**d**, AFM (**c**) and the corresponding height profile (**d**) of a monolayer ZIF film on HOPG, acquired from the indicated line in **c**. **e**, Monolayer and multilayer ZIF films on HOPG with discrete thicknesses as a function of synthesis time. The error bars in this figure represent the standard deviation of the difference in thickness within three measurements for each data point and the centre of each error bar represents the average thickness of the film. **f**, Optical image of a 2DZIF film on CVD graphene resting on a Cu foil. **g**, SEM image of a 2DZIF film on CVD graphene. The image was compiled by combining 1,155 (35 × 33) images by scanning the whole surface of the large sample.

The ZIF films were synthesized by immersing a substrate in an ultradilute precursor solution (≤2 mM Zn^2+^ and ≤16 mM 2-methylimidazole (2-mIm)) for a few minutes (Fig. 1a). The use of such ultradilute solutions for the growth of ZIF films has not been reported before (Fig. 1b and Supplementary Table 1). They were used here in an effort to suppress homogeneous nucleation in the bulk solution. With a diminished nuclei population in the bulk solution, the attachment of preformed nuclei to the substrate can be reduced or eliminated. This is expected to promote film growth by the assembly of molecular precursors on the substrate. Since this thin-film growth mode is anticipated to be sensitive to the type of substrate, we carried out synthesis using distinct substrates: (1) graphitic substrates with atomically smooth terrace such as highly oriented pyrolytic graphite (HOPG) or graphene, (2) Si/SiO_2_ wafer with a 300-nm-thick oxide layer, (3) single-crystal sapphire (Al_2_O_3_), (4) single-crystal quartz (SiO_2_) and (5) polycrystalline gold film.

ZIF films prepared on HOPG using a growth solution of 1 mM Zn^2+^ and 8 mM 2-mIm and reaction time of 5 min were examined by optical and scanning electron microscopy (SEM) (Supplementary Fig. 1). A sharp change in contrast was observed at the air/precursor solution interface beyond which the film had a uniform contrast, indicating that the film was smooth, continuous and macroscopically uniform. Atomic force microscopy (AFM) imaging near the interface confirmed that the ZIF film is indeed continuous and has a thickness of approximately 2 nm (Fig. 1c,d). When the synthesis time was reduced to 2 min, we observed a sub-monolayer film with micrometre-sized domains (Supplementary Fig. 2). The domains were faceted and had a thickness of 2 nm, consistent with the thickness of the continuous film, indicating that the film is crystalline consisting of micrometre-sized grains. We could obtain 4- and 6-nm-thick films by increasing the growth time from 5 min to 10 and 15 min, respectively (Fig. 1e and Supplementary Fig. 3a–f). Further increasing the growth time to 20 min did not lead to a thicker film, indicating precursor depletion (Supplementary Fig. 3g–i). Thicker (8 nm) films could be obtained by doubling the precursor concentration (Supplementary Fig. 3j–l). A discrete, 2 nm increase in film thickness further suggests a crystalline order. A fitting of film thickness with the number of probable layers yielded a monolayer thickness of 2 nm (Fig. 1e). Macroscopically large ZIF films spanning several centimetres in width could be obtained on chemical vapour deposition (CVD)-derived graphene film resting on a Cu foil (Fig. 1f,g).

**Fig. 2:**
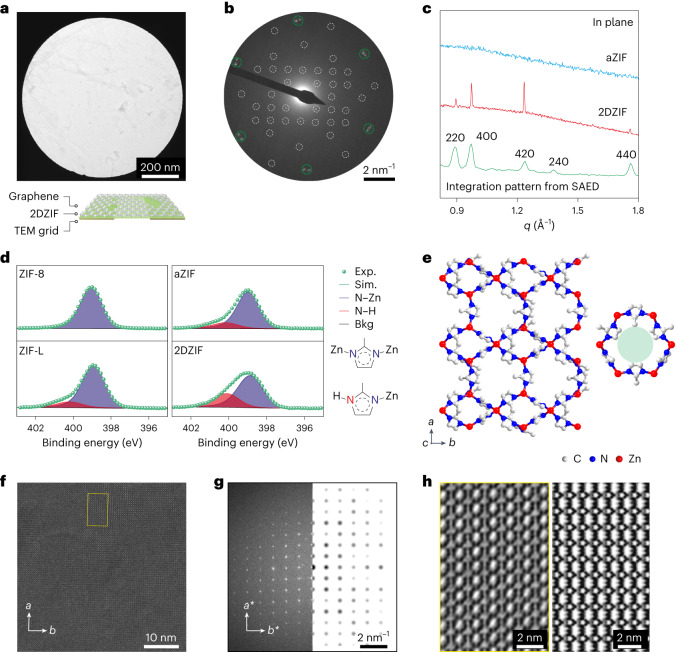
Structure determination of 2DZIF films. **a**, Bright-field TEM image of the 2DZIF film supported on suspended graphene. **b**, Corresponding SAED pattern. The pattern from graphene is identified with green circles and those from 2DZIF, with white circles. **c**, In-plane GIXRD data from an aZIF film (top) and a 2DZIF film (middle) prepared on Si/SiO_2_ and graphene/Si/SiO_2_, along with a radially integrated trace (bottom) of the SAED pattern shown in **b**. **d**, N1*s* XPS spectra from ZIF-8, ZIF-L, aZIF and 2DZIF films. The N–Zn and N–H coordination environments are shown on the right. **e**, DFT-relaxed structure of the 2DZIF and a visualization of 6-MR (right). **f**, High-resolution TEM image of the 2DZIF film lying flat on the *hk*0 plane, resting on suspended graphene. **g**, Corresponding Fourier transform compared with the simulated diffraction pattern from the proposed structure oriented along the *c*-out-of-plane direction. **h**, Contrast-transfer-function-corrected image of the highlighted area in **f** based on a defocus value of –130 nm analysed from the Thon rings in the Fourier transform pattern (left). Simulated projected potential map along the [001] direction of 2DZIF (right).

Graphene-supported ZIF film could be suspended on a holey transmission electron microscopy (TEM) grid (Fig. 2a). The film was devoid of large crystals and appeared uniform. The selected area electron diffraction (SAED) data from a micrometre-sized area yielded three sets of diffraction pattern (Fig. 2b). The first two sets (Fig. 2b, green circles) had six-fold symmetry originating from two slightly misoriented (by 3.0°) grains of graphene, whereas the last set had two-fold symmetry and belonged to a single grain of ZIF (white circles), confirming that ZIF prepared on graphene was crystalline with grains at least a micrometre in size, consistent with the AFM-based imaging of grains in the sub-monolayer film (Supplementary Fig. 2). Hereafter, the ZIF films on graphitic substrates are referred to as 2DZIF. The fact that a single 2DZIF grain could grow over two slightly misoriented graphene grains indicates that the growth could accommodate a small mismatch in its registry with the substrate. Diffraction pattern from 2DZIF, typically representing a single grain, was observed from every single spot over a large area. Based on the diffraction pattern, *a* and *b* lattice parameters of 2.4 and 2.0 nm, respectively, could be fitted (Supplementary Note 1 and Supplementary Tables 2 and 3).

We carried out synchrotron grazing-incidence X-ray diffraction (GIXRD) of a 10-nm-thick ZIF film on graphene resting on a Si/SiO_2_ wafer (Supplementary Figs. 4–6). The in-plane GIXRD pattern revealed sharp diffraction peaks, consistent with the peak positions obtained by the radial integration of the SAED pattern (Fig. 2c and Supplementary Table 3), confirming that the film formed on the graphitic substrate exhibits crystalline order. An analysis of the orientation of 2DZIF grain over graphene by SAED showed that the 2DZIF films crystallized, maintaining a fixed set of orientation with graphene (Supplementary Fig. 7, Supplementary Note 1 and Supplementary Table 2) and indicating the role of substrate registry in 2DZIF crystallization. This was further confirmed by synthesis on other crystalline substrates. Preferentially oriented 2DZIF films were achieved on single-crystal sapphire (Al_2_O_3_), single-crystal quartz (SiO_2_) and polycrystalline Au film, as indicated by the GIXRD and SAED data (Supplementary Figs. 8–12, Supplementary Note 2 and Supplementary Tables 4 and 5). In contrast, we did not observe diffraction from the ZIF film prepared on an amorphous substrate (Si/SiO_2_ wafer), indicating that the film was amorphous (hereafter referred to as amorphous zeolitic imidazolate framework (aZIF) films; Supplementary Fig. 13 and Fig. 2c). The presence of the order and preferred orientation in the ZIF film when prepared over a crystalline substrate and the lack of an order when prepared over the amorphous substrate indicates a strong role of substrate registry in the formation of the ordered 2DZIF film (Supplementary Tables 2 and 5). The ordered substrate probably promotes an ordered assembly of the molecular precursors for 2DZIF.

X-ray photoelectron spectroscopy (XPS) of the 2DZIF and aZIF films was carried out to gain further insights into their coordination environments (Fig. 2d). The N1*s* XPS data of 2DZIF compared with those of ZIF-L layers and a prototypical non-layered ZIF (ZIF-8) revealed that both 2DZIF and ZIF-L yield two peaks (399.0 and 400.2 eV corresponding to N–Zn and N–H bonds, respectively) in contrast to a single peak (399.0 eV) from the ZIF-8 crystals. This is consistent with the presence of abundant surface terminations (N–H) in the 2DZIF layers. In comparison, the population of N–H species was substantially diminished for aZIF, indicating a non-layered amorphous structure. The Zn2*p* XPS spectra were similar for all the samples (Supplementary Fig. 14), indicating a similar coordination environment for Zn.

**Fig. 3:**
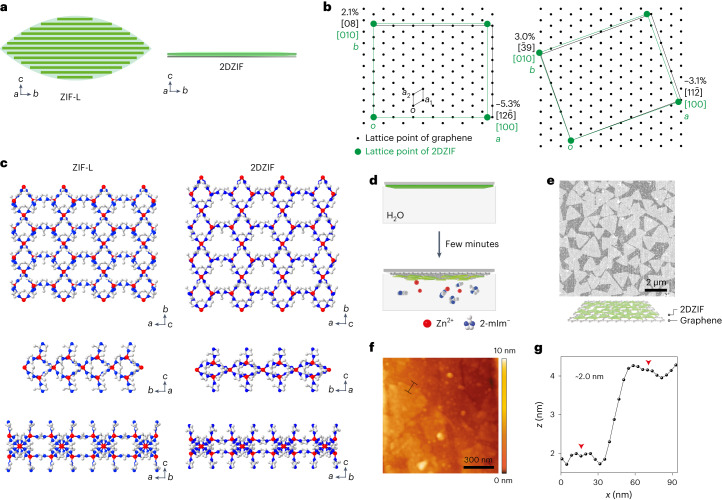
2DZIF structure and its relationship with ZIF-L. **a**, Schematic contrasting the arrangement of layers within a ZIF-L crystal with that of a monolayer 2DZIF. **b**, Registry between one unit cell of 2DZIF and a supercell of graphene based on SAED data (Supplementary Fig. 7). **c**, Structures of a ZIF-L layer (left) and 2DZIF (right) viewed along the [001], [100] and [010] directions. **d**, Schematic illustrating the etching of 2DZIF in water. **e**,**f**, SEM (**e**) and AFM (**f**) images of the triangular grains of 2DZIF obtained by a short etching in water. **g**, AFM height profile corresponding to the line in **f**.

To gain an insight into the structure of 2DZIF, structural relaxation based on DFT was carried out starting with the *a* and *b* lattice parameters obtained by SAED. The *c*-axis parameter was estimated by the AFM measurements (2 nm), and was subsequently relaxed by DFT calculations. The relaxed structure has an orthorhombic space group *Cmce* with the following structural parameters: *a* = 24.196 Å, *b* = 19.719 Å, *c* = 20.908 Å, *α* = 90°, *β* = 90° and *γ* = 90° (Supplementary Table 3). The layer in 2DZIF is composed of alternating 4-member-ring (MR) and 6-MR chains, whereas terminal 2-mIm linkers are present on both sides of the layer (Figs. 2e and 3c). The pore aperture of 2DZIF is constituted by 6-MR (Fig. 2e), which is attractive for gas separation.

Aberration-corrected high-resolution transmission electron microscopy (AC-HRTEM) imaging of the 2DZIF film suspended on a TEM grid was carried out along the [001] crystallographic direction (Fig. 2f). The imaging was carried out using a low-dose beam condition^41^ to minimize damage to the beam-sensitive 2DZIF lattice. Indeed, the obtained high-resolution TEM image revealed the high crystallinity of the 2DZIF film. The corresponding Fourier transform validated the *c*-out-of-plane orientation of the film and was consistent with the simulated electron diffraction pattern from a film lying flat along the same orientation (Fig. 2g). Projection along the *c*-out-of-plane axis from the contrast-transfer-function-corrected image revealed alternating chains of 4-MR and 6-MR (Fig. 2h, left), consistent with the simulated [001]-projected electrostatic potential map of 2DZIF structure obtained by DFT structural relaxation (Fig. 2h, right).

The registry or the lack of registry of 2DZIF with the underlying substrate plays an important role in determining its structure and morphology, especially when contrasted against the closely related material, namely, ZIF-L. Figure 3a highlights the morphological differences in ZIF-L and 2DZIF. Although the layers in ZIF-L and 2DZIF are stacked along the *c* axis, the former grows as leaf-shaped layered crystals (Supplementary Fig. 15), whereas the latter can form macroscopically uniform monolayer films. The unique leaf shape is formed because ZIF-L layers stack first with progressively increasing and then with progressively decreasing lateral size along the *b* axis. In contrast, as discussed before, registry with an underlying graphene substrate promotes the in-plane growth of the 2DZIF film (Fig. 3b, Supplementary Note 3 and Supplementary Fig. 7). Although both ZIF-L and 2DZIF have orthorhombic lattices, the unit-cell parameters of 2DZIF grown on graphene, obtained by DFT relaxation, are distinct from those of ZIF-L, where the latter has a substantially shorter parameter along the *b* axis (17.060 Å) compared with the former (19.719 Å; Fig. 3c and Supplementary Table 3).

The grains of 2DZIF could be visualized by the partial etching of 2DZIF films based on the well-documented dissolution of ZIFs in water (Fig. 3d)^42^. After partial dissolution, the grain shape was triangular with a lateral size of 1–2 µm (Fig. 3e), consistent with earlier observations of domains in the sub-monolayer film. The three sides of the triangular grains could be assigned to the (110), ($1\bar{1}0$) and (100) lattice planes, respectively, reported to be the minimum-surface-energy planes for ZIF layers (Supplementary Fig. 16)^43^. AFM images (Fig. 3f,g) confirmed that the grains have a uniform thickness of ~2 nm, consistent with the structure of 2DZIF. The SAED data of this sample showed the same pattern with 2DZIF, confirming that the triangular domains were indeed 2DZIF (Supplementary Fig. 17 and Supplementary Table 6).

## Gas separation performance of 2DZIF film

**Fig. 4:**
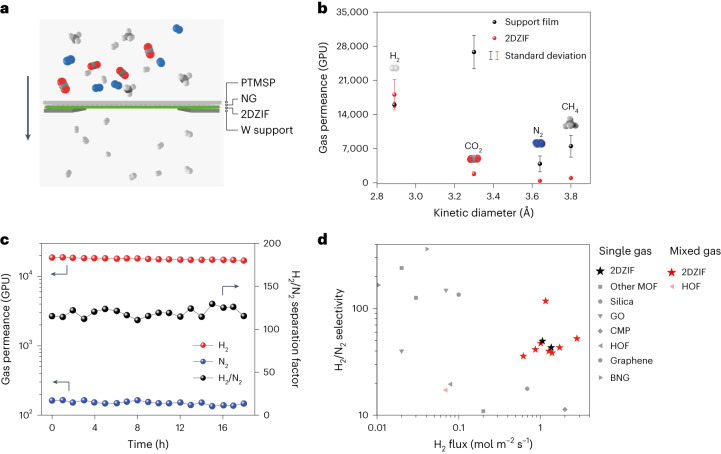
Gas separations of 2DZIF. **a**, Schematic of a 2DZIF film supported on NG reinforced with PTMSP. **b**, H_2_, CO_2_, N_2_ and CH_4_ permeances of the support film (PTMSP-reinforced NG) and the supported 2DZIF film. The error bar is the standard deviation from three samples and the centre of each error bar represents the average data from three samples (Supplementary Table 7). **c**, 2DZIF membrane separation performance for an equimolar H_2_/N_2_ mixed feed. **d**, Comparison of the H_2_/N_2_ separation performance of 2DZIF membranes with the state of the art (Supplementary Table 8). GO, CMP, HOF and BNG refer to graphene oxide, conjugated microporous polymers, hydrogen-bonded organic frameworks, and boron nitride and graphene nanosheet, respectively.

The 3.2 Å gap in 6-MR of 2DZIF is attractive for sieving H_2_ (kinetic diameter, 2.89 Å) from larger gas molecules such as CO_2_ (3.30 Å), N_2_ (3.64 Å) and CH_4_ (3.80 Å)^44,45^. The 2DZIF film is mechanically robust with Young’s modulus of 8.1 ± 2.1 GPa (Supplementary Fig. 18), comparable with that of the three-dimensional analogues^46^. Therefore, we probed the H_2_-sieving performance on the 2DZIF film. For this, the 2DZIF films were grown on nanoporous graphene (NG) (Supplementary Fig. 19) mechanically reinforced with a dense 250-nm-thick poly[1-(trimethylsilyl)propyne] (PTMSP) film (Supplementary Figs. 20 and 21), where the NG/PTMSP film acts as a support film (Fig. 4a). The pores in NG were intentionally designed to be large (1.8 ± 1.2 nm)^47^ to rule out any molecular sieving from NG and to allow the determination of H_2_ sieving from the 2DZIF film. The 2DZIF films, resting on a macroporous metal foil support (pore size, 5 µm; area, 1 mm^2^), exhibited a molecular cutoff for molecules larger than H_2_, indicating that gas transport was controlled by 6-MR of 2DZIF (Fig. 4b and Supplementary Figs. 22 and 23). The H_2_ permeance was large (>15,000 gas permeation units (GPU); 1 GPU = 3.35 × 10^−10^ mol m^−2^ s^−1^ Pa^−1^) similar to that from the support film (Supplementary Table 7 and Supplementary Fig. 22), indicating a negligible transport resistance from the 2DZIF layer.

When an equimolar H_2_:N_2_ mixture was probed with a feed pressure of 2 bar, a H_2_ permeance of 17,300 GPU with a H_2_/N_2_ separation factor of 115 could be obtained (Fig. 4c). Another membrane when tested under a high-pressure feed (8 bar), exhibited a high H_2_ flux of 2.8 mol m^−2^ s^−1^ and H_2_/N_2_ separation factor of 52 (Supplementary Fig. 24). This performance constitutes one of the best combinations of H_2_ flux and H_2_/N_2_ separation factor with the lowest thickness (Fig. 4d, Supplementary Figs. 25–27 and Supplementary Tables 8 and 9). Larger-area (centimetre-scale) 2DZIF membrane could also be prepared (Supplementary Fig. 28), owing to the highly uniform deposition of 2DZIF films on graphene (Fig. 1f,g). They also show attractive H_2_ permselective separation performance (Supplementary Table 10), in agreement with the smaller-area membranes (Supplementary Note 4).

We could obtain macroscopically smooth and uniform aZIF films with a thickness of 8–18 nm on Si/SiO_2_ wafers (Supplementary Fig. 13), which can find applications in resists for photolithography (Supplementary Figs. 29–33, Supplementary Table 11 and Supplementary Note 5)^48^.

The method reported here can be extended to other promising MOF structures. A 2D film of UiO-66-NH_2_ can also be deposited on graphene (Supplementary Fig. 34). It makes this approach reported here broad and interesting to develop a number of 2D MOF films in the future. In conclusion, we report the synthesis of ZIFs as macroscopically uniform amorphous and crystalline 2D films from an ultradilute solution. The 2DZIF film yields exceptional H_2_-sieving performance, owing to the ordered 2D structure with a high density of 6-MR hosting the H_2_-selective gap, making such a film the ultimate selective layer for membrane application. In the absence of substrate registry, ultrathin amorphous films are demonstrated, which are promising for advancing the limit of nanoscale patterning.

## Methods

### Chemicals

Zinc nitrate hexahydrate (Zn(NO_3_)_2_∙6H_2_O) was purchased from Sigma-Aldrich. Also, 2-mIm was obtained from Chemie Brunschwig AG. HCl (32 wt%) was purchased from Reactolab S.A. PTMSP was obtained from ABCR. FeCl_3_ (97%) and Na_2_S_2_O_8_ was bought from Sigma-Aldrich. Cu foil (50 mm, 99.9%) was purchased from Strem. Toluene (AR) and methanol (AR) were obtained from Fischer. All the chemicals were used without further purifications. Si/SiO_2_ wafers were purchased from University Wafer. Si/SiO_2_ wafer with single-layer graphene was bought from Ted Pella. HOPG (ZYA quality, GRAS/1.0 × 7.0 × 7.0) was purchased from ScanSens. Silicon nitride TEM supports (50 nm silicon nitride film on a 200 μm silicon frame with nine viewing windows, each 0.1 × 0.1 mm^2^) were purchased from Ted Pella. Sapphire (Al_2_O_3_) wafer (*Z* cut, 10 cm × 0.5 mm) was purchased from MTI. A TEM grid with 100-nm-thick *c*-out-of-plane-oriented gold film was purchased from Electron Microscopy Sciences. Single-crystal quartz (SiO_2_) wafer (*Z* cut, 10.0 mm × 10.0 mm × 0.5 mm) was bought from MSE Supplies.

### Characterizations and measurements

SEM measurements were performed on a Teneo SEM instrument operating at 1 kV. The powder X-ray diffraction data were collected at a Bruker D8 Discover diffractometer with a Lynxeye XE detector, operated at 40 kV and 400 mA under Cu Kα radiation (*λ* = 1.5406 Å) at ambient temperature and pressure. Bright-field TEM images and SAED images were obtained with a Talos F200X microscope operated at 200 kV. TEM images for patterns on silicon nitride were obtained on a Thermo Fisher TF30 TEM instrument operating at 300 kV.

Low-dose AC-HRTEM was performed on a Cs-corrected FEI G2 Titan^3^ 60-300 electron microscope at 300 kV, using a Gatan K2 direct-detection camera in the electron-counting mode. The AC-HRTEM images were acquired with the dose fractionation function, and each image stack is composed of 120 frames with 0.05 s exposure for each frame, with a total electron dose of ∼60 e^–^ Å^−2^. The raw image was denoised by using an average background subtraction filter. The contrast transfer function correction was performed based on the defocus value determined from the amorphous Thon rings in the Fourier transform, and the projected electrostatic potential was simulated by the QSTEM software (QSTEM v. 2.31). The simulated electron diffraction pattern was carried out using the single-crystal module of CrystalMaker software (version 9.0.1).

AFM images and modulus measurements were recorded on a Bruker MultiMode 8 AFM instrument. For modulus measurement, a Bruker Tap525A rectangular probe was used and calibrated with a standard sample of sapphire, polystyrene and HOPG. XPS data were obtained on an Axis Supra instrument (Kratos Analytical) using the monochromated K X-ray line of an aluminium anode. Synchrotron GIXRD data were obtained at beamline BM01, Swiss-Norwegian beamline (SNBL), at the European Synchrotron Radiation Facility (ESRF) at wavelengths of 0.683 and 0.960 Å.

The gas separation performance of all the membranes was recorded on a home-made permeation setup. The pressure on the feed side was maintained at 2–8 bar and on the permeate side at 1 bar during the measurements. All measurements were done after reaching the steady state with argon as the sweep gas. The membranes were sealed with a stainless-steel gasket. The composition of the permeate was analysed using an online Hiden Analytical HPR-20 mass spectrometer.

The permeances *J*_*i*_ of gas *i* was calculated as follows:

1 $${J}_{i}={X}_{i}/(A{\rm{\times }}\Delta {P}_{i}),$$

where *X*_*i*_ is the molar flow rate of component *i* across membrane area *A*, and ∆*P*_*i*_ are the transmembrane pressure difference for component *i*. The selectivity *α*_*ij*_ of two gases (*i* and *j*, where *i* is the faster permeating gas) was calculated as follows.

2 $${\alpha }_{{ij}}={J}_{i}/{J}_{j}$$

### Synthesis of 2DZIF/aZIF film

In a Petri dish containing 29 ml of Zn(NO_3_)_2_ aqueous solution at room temperature, the substrate (HOPG, graphene/Si/SiO_2_ for 2DZIF and Si/SiO_2_ for aZIF) was partially immersed. Then, 1 ml of 2-mIm aqueous solution was added. After a certain time, the substrate was removed to stop the reaction.

### Synthesis of 2DZIF membrane for gas separation

First, single-layer graphene was synthesized by using low-pressure CVD of methane on a copper foil following the literature^49^. Before the synthesis, the copper foil was annealed at 1,077 °C in a H_2_/Ar atmosphere for 60 min. Then, CO_2_ (100 ml min^–1^) and H_2_ (8 ml min^–1^) flow was successively introduced, each for 30 min, to remove the contaminations. At last, CH_4_ (24 ml min^–1^) and H_2_ (8 ml min^–1^) flows were used to grow single-layer graphene on the copper film for 30 min at a pressure of 460 mtorr.

After the synthesis of single-layer graphene, an O_2_ plasma (MTI Plasma Cleaner, EQ-PCE-3, 13.56 MHz, 17 W) was carried out to introduce nanopores for the application of a 2DZIF film as the selective layer in the membrane. Briefly, the atmosphere in the plasma chamber was exchanged by O_2_ flow to a pressure of around 50 mtorr. Then, a plasma was generated for 4 s to etch the single-layer graphene to obtain NG. After the plasma, a solution of PTMSP in toluene (1.25 wt%) was spin coated on NG at 1,000 r.p.m. for 30 s and 2,000 r.p.m. for 30 s. The sample was dried in ambient air at room temperature overnight. Then, the copper foil was etched by a combination of FeCl_3_ (0.5 M in water), HCl (0.1 M in water) and water. The floating graphene/PTMSP film was transferred on the surface of a 29 ml Zn(NO_3_)_2_ aqueous solution (2 mmol l^–1^) in a Petri dish, and an aqueous solution of 2-mIm (1 ml, 0.5 mol l^–1^) was added. After 2 min of reaction, the resulting 2DZIF/graphene/PTMSP film was transferred onto the substrate (for example, macroporous W support hosting 5 µm holes over a 1 mm^2^ area for the membranes) for further characterizations or applications. For the synthesis of centimetre-scale 2DZIF membrane, 1 wt% of Teflon AF in Galden perfluorinated fluid was spin coated on NG at 300 r.p.m. for 60 s and heated at 60 °C for 3 h. After that, the sample was put into a home-made membrane module (Supplementary Fig. 28) and Cu foil facing up. This allowed the etching of Cu foil in the membrane module by 10 wt% Na_2_S_2_O_8_ aqueous solution, exposing the NG surface for 2DZIF synthesis. Finally, the 2DZIF film was synthesized by exposing NG to growth solution for 10 min at room temperature (2 mM Zn(NO_3_)_2_ and 16 mM 2-mIm).

### Sample preparation of 2DZIF on graphene for AFM

For the AFM sample preparation of 2DZIF film on a graphene/PTMSP substrate, the 2DZIF/graphene/PTMSP film was transferred on a Si/SiO_2_ wafer with the 2DZIF layer facing the wafer. Then, the sample was annealed at 70 °C for 4 h to increase adhesion between the film and Si/SiO_2_ wafer. After that, the sample was immersed in toluene for 12 h to remove the PTMSP layer.

The AFM image of the 2DZIF film with triangular morphology after 5 min of water etching was directly collected on the film with the 2DZIF layer facing up. Specifically, the 2DZIF/graphene/PTMSP film with triangular morphology was first scooped by a glass slide with the 2DZIF layer facing the slide, and a Si/SiO_2_ wafer attached with double-sided carbon tape was pressed onto the PTMSP layer. As a result, the 2DZIF/graphene/PTMSP film was transferred onto the Si/SiO_2_ wafer, resulting in a 2DZIF layer facing up. The AFM measurement of 2DZIF film on HOPG, Si/SiO_2_ and graphene/Si/SiO_2_ was carried out directly on the sample without any treatment.

### Sample preparation for TEM

Similar to the sample preparation for AFM, a 2DZIF/graphene/PTMSP film was transferred on a TEM grid with the 2DZIF layer facing the TEM grid. Then, the sample was annealed at 70 °C for 4 h to increase adhesion between the film and TEM grid. After that, the sample was immersed in toluene for 12 h to remove the PTMSP layer.

For the electron patterning sample, aZIF layer was grown on silicon nitride supports. Before ZIF deposition, the silicon nitride supports were treated with oxygen plasma for 10 min (29.6 W, 400 mtorr oxygen pressure) in a plasma cleaner (Harrick Plasma) to improve the surface reactivity.

### Electron-beam patterning of aZIF films

For negative-tone patterning, aZIF films were exposed to an electron beam using a Thermo Fisher Helios G4 UC DualBeam microscope operating at 20 kV accelerating voltage and 400 pA beam current. The areal doses were 80 mC cm^–2^ for all the patterns. After exposure, the aZIF films were developed in deionized water for 24 h and blow dried in a stream of nitrogen gas.

For positive-tone patterning, the aZIF films were first placed in a 60 ml PFA vessel with a bed of 0.2 g of 4,5-dichloroimidazole and heated at 75 °C for 1.5 h. The 4,5-dichloroimidazole-treated aZIF was then exposed to an electron beam using a Thermo Fisher Helios G4 UC DualBeam microscope operating at 20 kV accelerating voltage and 100 pA beam current. The areal doses were 2 mC cm^–2^ for all the patterns. After exposure, the aZIF films were immersed in n-methyl-2-pyrrolidone (NMP) and acetone each for 10 s and blow dried in a stream of nitrogen gas.

### Spin coating of aZIF films on silicon wafers

Aqueous solutions of Zn(NO_3_)_2_ (4 mmol l^–1^) and 2-mIm (32 mmol l^–1^) were mixed in a T connector (0.5 mm inner diameter) at 1 ml min^–1^ injection rate using a two-channel syringe pump. The mixed solution was spin coated on silicon wafers and spinning at speeds between 500 and 2,000 r.p.m. for 1 min, followed by 10 s spinning at 2,000 r.p.m. to completely dry the film. The spin-coated films were exposed to an electron beam using line patterns of 100 nm width and 400 nm spacing and developed in deionized water for 24 h. The height of the line patterns were measured to determine the thickness of spin-coated films.

### Structural simulation

The simulation of the 2DZIF structure was carried out by the DFT calculations. At first, the reported ZIF-L structure was imported into forcite module in Materials Studio software (Accelrys) (v8.0.0.843) to calculate the initial structural model, and the unit cell was set to be orthorhombic with *a* = 24.0 Å, *b* = 20.0 Å, *c* = 20.0 Å, *α* = 90°, *β* = 90°, *γ* = 90°, whereas the connectivity was kept. The calculation task was geometry optimization. The quality was set to be fine, and the ‘smart’ algorithm was selected. After that, van der Waals DFT calculations were performed using the QUANTUM ESPRESSO package^50,51^. The Brillouin zone was sampled at the gamma point. An energy cutoff of 60 Ry was used for the plane-wave expansion of wavefunctions. A kinetic energy cutoff of 480 Ry on the charge was used together with ultrasoft pseudopotentials^52,53^. The relaxation was performed with the Perdew–Burke–Ernzerhof functional^54^. The system was relaxed to the lowest-energy configuration of atoms. The surface geometry had been optimized with the convergence thresholds of 1 × 10^−4^ Ry and 3.28 × 10^−3^ Ry Bohr^–1^ for the total energy and forces, respectively.

## Online content

Any methods, additional references, Nature Portfolio reporting summaries, source data, extended data, supplementary information, acknowledgements, peer review information; details of author contributions and competing interests; and statements of data and code availability are available at 10.1038/s41563-023-01669-z.

### Supplementary information

Supplementary Information Supplementary Figs. 1–34, Notes 1–5 and Tables 1–11.

Supplementary Data The CIF file of the simulated structure of 2DZIF.

## Data Availability

Data supporting the findings in the present work are available in the Article or its Supplementary Information. Additional data are available from the corresponding author upon request or available via Zenodo at 10.5281/zenodo.8212765.
