# Supplementary material for: Unit-cell-thick zeolitic imidazolate framework films for membrane application
Source: Nat Mater. 2023 Sep 21;22(11):1387–93. doi: 10.1038/s41563-023-01669-z (PMC10627807; doi:10.1038/s41563-023-01669-z)
Supplement: Supplementary file 1 — Supplementary Figs. 1–34, Notes 1–5 and Tables 1–11. [file 41563_2023_1669_MOESM1_ESM.pdf]

nature materials

Article

https://doi.org/10.1038/s41563-023-01669-z

Unit-cell-thick zeolitic imidazolate framework films for membrane application

In the format provided by the authors and unedited

Table of Contents

**Supplementary Table 1.** Comparison of synthesis conditions for ZIF-L and 2DZIF.	2
**Supplementary Table 2.** Comparison of experimental (Exp.) electron diffraction data and theoretical (Theor.) data.	2
**Supplementary Table 3.** Lattice parameters for 2DZIF@graphene extracted from SAED and GIXRD as well as relaxed DFT structure and compared to those of ZIF-L.	3
**Supplementary Table 4.** Comparisons of lattice parameters for *c*-orientated sapphire, quartz, gold and 2DZIF on different substrates extracted from SAED and GIXRD.	3
**Supplementary Table 5.** Comparison of experimental (Exp.) X-ray diffraction data and theoretical (Theor.) data.	3
**Supplementary Table 6.** Lattice parameters for 2DZIF@graphene compared to those after water etching.	5
**Supplementary Table 7.** Average single gas permeance and ideal selectivity of various membranes.	5
**Supplementary Table 8.** Gas permeance and ideal selectivity of our membranes compared to the state-of-art membranes in the literature.	6
**Supplementary Table 9.** Thickness and separation performance of 2DZIF membranes compared to the state-of-art MOF membranes in the literature.	7
**Supplementary Table 10.** Single gas permeance and ideal selectivity of centimeter-scale membrane.	8
**Supplementary Table 11**. Sensitivity of representative commercial and metal-containing resists for electron beam lithography.	9
**Supplementary Note 1.** Index of electron diffraction data from 2DZIF@substrate films.	10
**Supplementary Note 2.** GIXRD measurement and analyses of 2DZIF@substrate films.	11
**Supplementary Note 3.** Calculation of lattice mismatch between 2DZIF and underlying substrate.	14
**Supplementary Note 4.** Calculation of intrinsic performance of 2DZIF layer in 1 cm scale membrane.	14
**Supplementary Note 5.** Syntheses and patterning application of amorphous ZIF films.	15
**Supplementary data for the structural characterizations of 2DZIF films**	17
**Supplementary data for the gas separation performances of 2DZIF films**	33
**Supplementary data for the e-beam patterning of aZIF films**	39
**References**	43

Supplementary Table 1. Comparison of synthesis conditions for ZIF-L and 2DZIF.

	$Zn(NO_3)_2$ (Wt.%)	2-mIm (Wt.%)	H_2O (Wt.%)	Temperature	Time	Ref.
1	1.42	3.13	95.45	RT	4 h	14
2	0.72	1.59	97.69	RT	4 h	15
3	0.065	8.21	91.725	RT	14 h	16
4	0.36	3.56	96.08	RT	4 h	17
5	1.38	3.26	95.36	RT	1 h	18
6	0.72	1.59	97.69	RT	4 h	19
7	0.83	1.82	97.35	30 °C	1 h	20
8	1.26	5.56	93.18	RT	30 min	21
9	0.059	0.13	99.811	RT	≤15 min	This work

Supplementary Table 2. Comparison of experimental (Exp.) electron diffraction data and theoretical (Theor.) data.

Sample	*(hkl)*	*d* spacing (Å)		Angle (°)	
		Exp.	Theor.	Exp.	Theor.
Graphene	(10)	2.1	2.13	-	-
	(01)	2.1	2.13	120 (01)/(10)	120 (01)/(10)
2DZIF@Graphene	(020)	9.7	9.65	-	-
	(110)	15.1	15.06	40 (110)/(020)	38.7 (110)/(020)
2DZIF (Graphene, water-etched)	(020)	9.8	9.65	-	-
	(110)	15.0	15.06	41 (110)/(020)	38.7 (110)/(020)
Au	(200)	2.1	2.04	-	-
	(020)	2.1	2.04	90 (020)/(200)	90.0 (020)/(200)
2DZIF@Au	(110)	14.2	14.14	-	-
	($\bar{1}10$)	14.2	14.14	90 ($\bar{1}10$)/(110)	90.0 ($\bar{1}10$)/(110)

Supplementary Table 3. Lattice parameters for 2DZIF@graphene extracted from SAED and GIXRD as well as relaxed DFT structure and compared to those of ZIF-L.

	2DZIF@Graphene			ZIF-L
	ED	GIXRD	DFT	-
a (Å)	24.2	24.4	24.196	24.119
b (Å)	20.1	19.3	19.719	17.060
c (Å)	-	-	20.908	19.739

Supplementary Table 4. Comparisons of lattice parameters for *c*-orientated sapphire, quartz, gold and 2DZIF on different substrates extracted from SAED and GIXRD.

	Graphene	2DZIF @Graphene	*c*-out-of-plane orientated Sapphire	2DZIF @Sapphire	*c*-out-of-plane orientated Quartz	2DZIF @Quartz	*c*-out-of-plane orientated Au	2DZIF@ Au
		GIXRD	-	GIXRD	-	GIXRD	-	ED
a (Å)	2.46	24.4	4.79	24.1	4.91	23.9	4.08	21.3
b (Å)	2.46	19.3	4.79	20.1	4.91	19.6	4.08	18.9

Supplementary Table 5. Comparison of experimental (Exp.) X-ray diffraction data and theoretical (Theor.) data.

Sample	*(hkl)*	*q* ($Å^{-1}$)	*d* spacing (Å)		Angle (°)	
		Exp.	Exp.	Theor.	Exp.	Theor.
Sapphire	(006)	4.30	1.47	1.50	-	-
	(104)	2.88	2.18	2.16	23 (104)/(006)	21.5 (104)/(006)
	(018)	2.50	2.52	2.55	41 (018)/(006)	39.0 (018)/(006)
2DZIF@ Sapphire	(003)	0.90	6.98	6.97	-	-
	($\bar{1}$33)	1.31	4.79	4.74	47 ($\bar{1}$33)/(003)	47.2 ($\bar{1}$33)/(003)
	($\bar{2}$03)	1.03	6.10	6.03	28 ($\bar{2}$03)/(003)	30.0 ($\bar{2}$03)/(003)
	($\bar{1}$05)	1.5	4.19	4.12	10 ($\bar{1}$05)/(003)	9.8 ($\bar{1}$05)/(003)
	(105)	1.5	4.19	4.12	10 (105)/(003)	9.8 (105)/(003)
	(204)	1.3	4.83	4.79	22 (204)/(003)	23.5 (204)/(003)
	(103)	0.93	6.75	6.69	18 (103)/(003)	16.1 (103)/(003)
	(312)	1.05	5.98	6.07	55 (312)/(003)	54.5 (312)/(003)

	(112)	0.73	8.60	8.65	30<br>(112)/(003)	34.1<br>(112)/(003)
	(401)	1.1	5.71	5.79	72<br>(401)/(003)	74.0<br>(401)/(003)
	(503)	1.55	4.05	3.96	53<br>(503)/(003)	55.3<br>(503)/(003)
	(012)	0.68	9.24	9.27	25<br>(012)/(003)	27.5<br>(012)/(003)
	$(\bar{1}\bar{2}1)$	0.75	8.37	8.48	63<br>$(\bar{1}\bar{2}1)$/(003)	66.1<br>$(\bar{1}\bar{2}1)$/(003)
	$(\bar{3}01)$	0.82	7.66	7.50	70<br>$(\bar{3}01)$/(003)	69.0<br>$(\bar{3}01)$/(003)
	$(\bar{3}00)$	0.8	7.85	8.03	90<br>$(\bar{3}00)$/(003)	90<br>$(\bar{3}01)$/(003)
	$(\bar{5}03)$	1.55	4.05	3.96	53<br>$(\bar{5}03)$/(003)	55.3<br>$(\bar{5}03)$/(003)
	$(\bar{5}22)$	1.55	4.05	4.01	65<br>$(\bar{5}22)$/(003)	67.4<br>$(\bar{5}22)$/(003)
	$(\bar{6}01)$	1.58	3.97	3.94	80<br>$(\bar{6}01)$/(003)	79.2<br>$(\bar{6}01)$/(003)
	$(\bar{4}05)$	1.86	3.38	3.43	36<br>$(\bar{4}05)$/(003)	34.8<br>$(\bar{4}05)$/(003)
Quartz	(012)	2.73	2.30	2.28	-	-
	(102)	2.73	2.30	2.28	33<br>(102)/(012)	31.0<br>(102)/(012)
2DZIF@ Quartz	(003)	0.90	6.98	6.97	-	-
	(113)	0.99	6.34	6.33	-	-
2DZIF@ Graphene	(220)	0.86	7.31	7.55	-	-
	(400)	1.01	6.21	6.10	-	-
	(420)	1.24	5.07	5.16	-	-
	(440)	1.70	3.70	3.78	-	-

Supplementary Table 6. Lattice parameters for 2DZIF@graphene compared to those after water etching.

	2DZIF@Graphene	2DZIF@Graphene after partial etching in water
	ED	ED
a (Å)	24.2	24.1
b (Å)	20.1	20.1

Supplementary Table 7. Average single gas permeance and ideal selectivity of various membranes.

	H_2 permeance (GPU)	CO_2 permeance (GPU)	N_2 permeance (GPU)	CH_4 permeance (GPU)	H_2/CO_2	H_2/N_2	H_2/CH_4
PTMSP	22330±1180	39850±210	5490±420	8880±430	0.6	4.1	2.5
NG/PTMSP (support film)	15970±550	26800±3410	3890±1600	7500±2240	0.6	4.1	2.1
2DZIF supported on NG/PTMSP	18070±3130	1840±420	390±100	980±40	9.8	46.3	18.4

Supplementary Table 8. Gas permeance and ideal selectivity of our membranes compared to the state-of-art membranes in the literature.

	Name	Feed pressure (bar)	Single gas			Mixture gas			Ref.
			H_2 permeance (GPU)	H_2 flux ($mol \cdot m^{-2} \cdot s^{-1}$)	H_2/N_2	H_2 permeance (GPU)	H_2 flux ($mol \cdot m^{-2} \cdot s^{-1}$)	H_2/N_2	
MOF	ZIF-8	2	6000	0.40	6.5	6000	0.40	6.5	22
	CuBTC/MIL-100	2	284	0.02	240.4	-	-	-	23
	ZIF-L	2	582	0.04	8.1	530	0.04	7.7	24
	Vapor phase ligand treatment ZIF-8	2	448	0.03	126	-	-	-	25
Silica	Si	2	1493	0.10	135	-	-	-	26
Zeolite	SAPO-34	2.3	96	0.01	7.4	-	-	-	27
GO	SOD/GO	2	1194	0.02	40	-	-	-	28
CMP	CMT	2	29786	2.00	11.3	-	-	-	29
HOF	UPC-HOF-6-120	2.2	1045	0.08	19.5	890	0.07	17.2	30
Graphene	Nanoporous graphene	1.5	13731	0.69	17.7	-	-	-	49 in main text
BNG	BNG (7:25)	1	58.3	0.002	20.7	-	-	-	31
	BNG (7:10)	1	1150.5	0.04	375.1	-		-	
	BNG (7:5)	1	290.2	0.01	171.1	-	-	-	
2DZIF	2DZIF	2	20280	1.36	43.1	17300	1.16	117.8	This work
	2DZIF	2	15850	1.06	49.5	9300	0.62	35.8	
	2DZIF	3	-	-	-	8680	0.87	41.3	
	2DZIF	4	-	-	-	7560	1.01	47.3	
	2DZIF	5	-	-	-	7570	1.27	39.8	
	2DZIF	6	-	-	-	6920	1.39	38.4	
	2DZIF	7	-	-	-	7320	1.72	43.1	
	2DZIF	8	-	-	-	10450	2.80	52.3	

GO: graphene oxide, CMP: conjugated microporous polymers, HOF: hydrogen-bonded organic frameworks, BNG: boron nitride and graphene nanosheet

Supplementary Table 9. Thickness and separation performance of 2DZIF membranes compared to the state-of-art MOF membranes in the literature.

MOF	Thickness (nm)	H_2 flux ($mol \cdot m^{-2} \cdot s^{-1}$)	H_2/N_2 selectivity	Separation index	Ref.
ZIF-8	6000	0.24	3.2	0.5	32
	2000	0.15	1.6	0.1	22
	2500	0.05	10.6	0.5	34
	35000	0.02	12.4	0.2	35
	25000	0.02	5.3	0.1	36
	500	0.83	15.5	12.0	23 in main text
	1500	0.10	10.9	1.0	22
	620	0.04	-	-	37
	1100	0.004	16.8	0.1	38
	2000	5.73	15.4	82.5	39
	2000	0.04	11.1	0.4	40
	500	0.002	10.0	0.0	41
	8000	0.10	7.9	0.7	42
	200	2.05	9.7	17.8	43
	2600	0.003	-	-	44
	40000	0.01	22.7	0.2	45
	130	0.21	20.0	3.9	46
	200	2.87	18.0	48.8	47
	17	2.15	15.1	30.4	48
	12000	0.01	-	-	49
	20000	0.01	10.0	0.1	50
	30000	0.01	11.6	0.1	51
	20000	0.02	17.6	0.4	52
Cu-BTC	43000	0.85	5.9	4.1	53
$Zn_2(bIm)_4$	50	0.03	66.6	2.1	54
ZIF-8/GO	100	0.22	4.6	0.8	55
Y-fum-fcu-MOF	85	0.003	7.3	0.0	56
2DZIF	2	1.36	43.1	57.2	

	2	1.06	49.5	51.5	This work
	2	1.16	117.8	135.4	
	2	0.62	35.8	21.7	
	2	0.87	41.3	35.2	
	2	1.01	47.3	46.9	
	2	1.27	39.8	49.2	
	2	1.39	38.4	52.0	
	2	1.72	43.1	72.3	
	2	2.80	52.3	143.7	

Supplementary Table 10. Single gas permeance and ideal selectivity of centimeter-scale membrane.

	H_2 permeance (GPU)	CO_2 permeance (GPU)	N_2 permeance (GPU)	CH_4 permeance (GPU)	H_2/CO_2	H_2/N_2	H_2/CH_4
Support film (NG/Teflon AF)	2450	2290	610	390	1.1	4.0	6.3
2DZIF on support film	2180	340	110	80	6.4	19.8	27.3
Intrinsic performance of 2DZIF layer*	19781	399	134	101	50	148	195

*Intrinsic performance of 2DMOF layer is calculated based on the resistance-in-series model (see Supplementary Note 4).

Supplementary Table 11. Sensitivity of representative commercial and metal-containing resists for electron beam lithography.

Resist category	Material	Resist tone	Sensitivity (mC/cm^2)	Ref.
Polymer	PMMA	Positive	~ 0.2	Commercial
Silicon-oxo cage	HSQ	Negative	~ 0.5	Commercial
Metal-oxo cage	HafSOx	Negative	0.488	57
Metal-based cluster	Zn-mTA	Negative	0.1	58
Metal-containing polymer	PFpP	Negative<br>Positive	12 (negative)<br>10 (positive)	59
MOF	ZIF-71 ($Zn(dcIm)_2$)	Positive	1	60
MOF	aZIF	Negative<br>Positive	80 (negative)<br>2 (positive)	This work

PMMA: Poly(methyl methacrylate); HSQ: Hydrogen silsesquioxane.

Supplementary Note 1. Index of electron diffraction data from 2DZIF@substrate films.

Both electron diffraction data of 2DZIF@Graphene and 2DZIF@Au were indexed by the same way, where *d* spacing and angle of/between each diffraction dot were read and compared to theoretical data. Thus, according to the index, the orientations between 2DZIF and substrates were identified.

Theoretical *d* spacing value were calculated by the following equations,

For graphene, which are two-dimensional hexagonal system,

$$\frac{1}{d^2} = \frac{3}{2}\left(\frac{h^2 - hk + k^2}{a^2}\right)$$

For Au, which is cubic system,

$$\frac{1}{d^2} = \frac{h^2 + k^2 + l^2}{a^2}$$

Theoretical angles between lattice planes were calculated by the following equations,

For graphene, which is two-dimensional hexagonal system,

$$\cos\Phi = \frac{h_1 h_2 + k_1 k_2 - \frac{1}{2}(h_1 k_2 + h_2 k_1)}{\sqrt{(h_1^2 + k_1^2 + h_1 k_1)(h_2^2 + k_2^2 + h_2 k_2)}}$$

For Au, which is cubic system,

$$\cos\Phi = \frac{h_1 h_2 + k_1 k_2 + l_1 l_2}{\sqrt{(h_1^2 + k_1^2 + l_1^2)(h_2^2 + k_2^2 + l_2^2)}}$$

The equations for the calculations of theoretical *d* spacing value and angles between lattice planes of 2DZIF were the same as that in XRD data.

Supplementary Note 2. GIXRD measurement and analyses of 2DZIF@substrate films.

For the GIXRD measurement, 2DZIF films grown on different substrates were prepared by the same synthesis condition, which was 2 mM Zn^{2+}, 16 mM 2-mIm, and reaction time of 2 min. All data were collected at beamline BM01, Swiss-Norwegian beamline (SNBL) at the European Synchrotron Radiation Facility (ESRF) with a multipurpose PILATUS2M detector[1]. The wavelength for the data of 2DZIF@Graphene was 0.683 Å, while it was 0.960 Å for the data of both 2DZIF@Sapphire and 2DZIF@Quartz, respectively. The X-ray beam was always horizontally shading at the samples during testing. The size of X-ray beam was 200 μm × 80 μm.

i. Alignment of X-ray beam

Before the measurement, standard sample LaB_6 was used to align the X-ray beam. Then each sample was mounted horizontally and its position was aligned to make sure X-ray was half-stopped by the sample by a combination of ω-scan and z-scan.

ii. Alignment of measurement geometry

Before the collection of diffraction data, a further fine ω-scan was carried out first to collect a series of diffraction data, followed by checking the diffraction and looking for the best measurement geometry for the collection of diffraction data.

For the alignment of measurement geometry of 2DZIF@Graphene, a series of diffractions by ω-scans of the sample with each step of 0.01° and 20 s were first collected from -1° to 2° and analyzed to find the best diffraction geometry, where ω of 0.04° was found to be the best. The distance from the sample to the detector was 200 mm.

In contrast, for the alignment of measurement geometry of 2DZIF@Sapphire and 2DZIF@Quartz, due to the single crystal nature of the sapphire (Al_2O_3) and quartz (SiO_2) substrates and much smaller lattices of them compared to 2DZIFs, two ω-scans of the samples were performed for 2DZIFs and substrates, respectively. Specifically, similar to that of 2DZIF@Graphene, a series of diffractions of ω-scans of the sample with each step of 0.01° and 20 s were first collected from -1° to 2° for 2DZIF films, which led to the same measurement geometry compared to that of 2DZIF@Graphene. Meanwhile, single-crystal diffractions of ω-scans of the samples with each step of 1° and 10 s were collected from -5° to 20°. In result, the ω for sapphire and quartz were set to be 15° and 10°, respectively. The distance from the sample to the detector was 200 mm and 100 mm for sapphire and quartz, respectively.

iii. Collection of diffraction data

For the measurement of 2DZIF@Graphene, only the diffraction data of 2DZIF was collected where grazing incidence was applied at 0.04° to collect the diffraction of 2DZIF@Graphene, and collecting time was 60 s, with the distance of 200 mm from the sample to the detector.

For the measurements of 2DZIF@Sapphire and 2DZIF@Quartz samples. Both the diffraction data from 2DZIF and substrates were collected. Grazing incidence was applied at 0.04° to collect the diffraction of 2DZIF@Sapphire and 2DZIF@Quartz, and collecting time was 60 s, with the distance of 200 mm from the sample to the detector. Single-crystal X-ray diffractions were performed to collect the diffraction of substrates with ω of 10° and 15° for Sapphire and Quartz, respectively. Collecting time was 20 s, and the distance from the sample to the detector was 200 mm and 100 mm for sapphire and quartz, respectively.

iv. Analyses of X-ray diffraction data.

For the index of diffraction data, the q value and angle between each diffraction dot were read first, which provided corresponding d spacing value and angle. Then, theoretical d spacing value and angles of all lattice planes of the samples were calculated. Last, comparisons of experimental data with theoretical data were made and corresponding Miller indices was confirmed. Thus, according to this index, the orientation of sample was identified.

Experimental q value was calculated by the following equation,

$$q = \frac{4\pi\sin(\theta)}{\lambda}$$

Experimental d spacing value was calculated by the following equation,

$$d = \frac{\lambda}{2\sin(\theta)} = \frac{2\pi}{q}$$

Theoretical d spacing value were calculated by the following equations,

For sapphire and quartz, which are trigonal system,

$$\frac{1}{d^2} = \frac{4}{3}\left(\frac{h^2 + hk + k^2}{a^2}\right) + \frac{l^2}{c^2}$$

For 2DZIF, which is orthorhombic system,

$$\frac{1}{d^2} = \frac{h^2}{a^2} + \frac{k^2}{b^2} + \frac{l^2}{c^2}$$

Theoretical angles between lattice planes were calculated by the following equations,

For sapphire and quartz, which are trigonal system,

$$\cos\Phi = \frac{h_1h_2 + k_1k_2 + \frac{1}{2}(h_1k_2 + h_2k_1) + \frac{3a^2}{4c^2}l_1l_2}{\sqrt{(h_1^2 + k_1^2 + h_1k_1 + \frac{3a^2}{4c^2}l_1^2)(h_2^2 + k_2^2 + h_2k_2 + \frac{3a^2}{4c^2}l_2^2)}}$$

For 2DZIF, which is orthorhombic system,

$$\cos\Phi = \frac{\frac{h_1h_2}{a^2} + \frac{k_1k_2}{b^2} + \frac{l_1l_2}{c^2}}{\sqrt{(\frac{h_1^2}{a^2} + \frac{k_1^2}{b^2} + \frac{l_1^2}{c^2})(\frac{h_2^2}{a^2} + \frac{k_2^2}{b^2} + \frac{l_2^2}{c^2})}}$$

Supplementary Note 3. Calculation of lattice mismatch between 2DZIF and underlying substrate.

The lattice mismatch is calculated by the following equation,

$$\text{Lattice mismatch} = \frac{d_{film} - d_{substrate}}{d_{substrate}} * 100\%$$

Based on the results of electron diffractions and GIXRD data, where lattice parameter of 2DZIF on different substrates and orientation between lattices of 2DZIF and substrates are indexed first for each case. Since the lattice parameter of 2DZIF is much larger than that from substrates, superlattice of the substrate (supercell) is used to compare one lattice of 2DZIF in the calculation depending on the difference of the lattice 2DZIF and the substrate.

Supplementary Note 4. Calculation of intrinsic performance of 2DZIF layer in 1 cm scale membrane.

The resistance (R_i) of gas permeance membrane is defined as,

$$R_i = \frac{L}{P_i \times A} = \frac{1}{J_i \times A}$$

Where L is the thickness of membrane, P_i is permeability of the membrane material to gas i, J_i is permeance of the membrane material to gas i, and A is the effective area of membrane.

The 2DZIF membrane is composed of two parts, supportive NG/Teflon layer and selective 2DZIF layer, that means the total resistance (R_{total}) can be expressed as a combination of resistance from supportive NG/Teflon layer, $R_{support}$, and selective 2DZIF layer, R_{2DZIF},

$$R_{total} = R_{support} + R_{2DZIF}$$

According to the definition of resistance (R_i) of gas permeance membranes, we will have

$$\frac{1}{J_{total}} = \frac{1}{J_{support}} + \frac{1}{J_{2DZIF}}$$

Where J_{total}, $J_{support}$ and J_{2DZIF} are permeance of total membrane, support layer and 2DZIF layer, respectively[1].

Take H_2 permeance data from Supplementary Table 10 for example,

$$\frac{1}{2180(GPU)} = \frac{1}{2450(GPU)} + \frac{1}{J_{2DZIF}}$$

The above results in the intrinsic H_2 permeance of 2DZIF $J_{2DZIF} = 19781$ GPU.

Supplementary Note 5. Syntheses and patterning application of amorphous ZIF films.

We could obtain macroscopically smooth, continuous, and uniform aZIF films on Si/SiO_2 wafers. AFM of one of these films, prepared using 2 mM Zn^{2+} and 16 mM 2-mIm and deposition time of 10 s, indicated that the film is smooth with thickness near 8 nm (Supplementary Fig. 13c-e). Ellipsometry of several ZIF films on Si/SiO_2 wafers, prepared by varying the synthesis time, indicated that the film thickness could be tuned in the range of 8-18 nm consistent with the corresponding AFM data (Supplementary Fig. 13).

Amorphous MOFs exhibit unique physical and chemical properties due to the absence of anisotropy and crystalline grains[2]. On one hand, they may not have the well-defined pore structures of crystalline MOFs required for certain molecular sieving applications, but at the same time, they do not exhibit grain boundaries and structural anisotropies of crystalline MOFs, which can create film non-uniformities. A potential use of organic-inorganic films is in next generation resists for photolithography in place of currently used polymeric resists, and, for this application, MOF-inspired metal-organic clusters have been proposed for high resolution patterning (ref. 13 in main text).

As a demonstration of the potential of our deposition method in this emerging application, an aZIF film was deposited on a silicon nitride support and subsequently exposed to a direct-write electron beam using 1:1 line- and space-patterns ranging from 10 to 40 nm in line width (or half pitch) (Supplementary Fig. 29a). The aZIF films behave similarly to ZIF-L crystals, for which e-beam treatment can induce contrast in water dissolution behavior based on framework densification and disintegration of the ligand molecular structure[3-5]. After development in water, the irradiated area was preserved while the non-irradiated area was dissolved (Supplementary Fig. 29b), confirming aZIF as a negative-tone resist. The thickness of the remaining aZIF structure was determined to be ~25 nm by AFM (Supplementary Fig. 29c and 29d). The resolution of the resulting pattern, as exemplified by the well-resolved lines at 20 nm half pitch, is comparable to the state-of-the-art metal-containing resists[6], which are an emerging class of material that hold promise in extreme ultraviolet lithography and electron beam lithography[7-10].

aZIF can also be patterned in positive-tone mode by a vapor phase ligand pretreatment. The as-deposited aZIF is exposed to the sublimated vapor of 4,5-dichloroimidazole (dcIm) at 75 °C for 1.5 h, during which the aZIF matrix is partially exchanged or infiltrated with dcIm ligand. The dcIm-treated film is then exposed to a direct-write electron beam. After development in

organic solvents, the irradiated area is removed while non-irradiated area is preserved, which showed similar sensitivity compared to the reported data (Supplementary Fig. 30 and Supplementary Table 11). Furthermore, to improve compatibility with microfabrication processes, aZIF films are spin-coated on silicon wafers, and their thicknesses can be controlled by spin speed (Supplementary Fig. 31-33). The simple fabrication for ultrathin ZIF films reported in this study could accelerate the development of new ZIF-based resist materials for lithographic applications[11-13].

Supplementary data for the structural characterizations of 2DZIF films

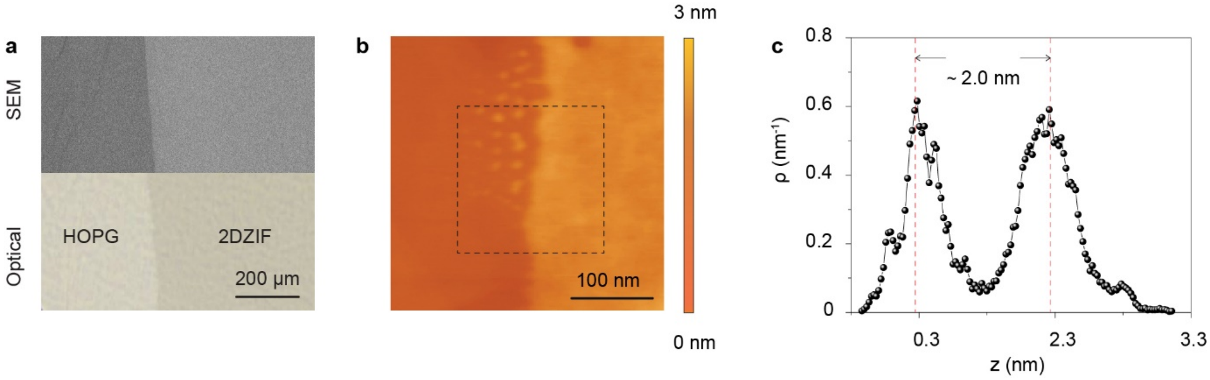

Supplementary Fig. 1. SEM and optical images (**a**), and AFM image (**b**) and height distribution (**c**) from square area labelled in (**b**) of a 2DZIF film on HOPG. Reaction condition, 1 mM Zn^{2+}; 8 mM 2-mIm; reaction time of 5 min.

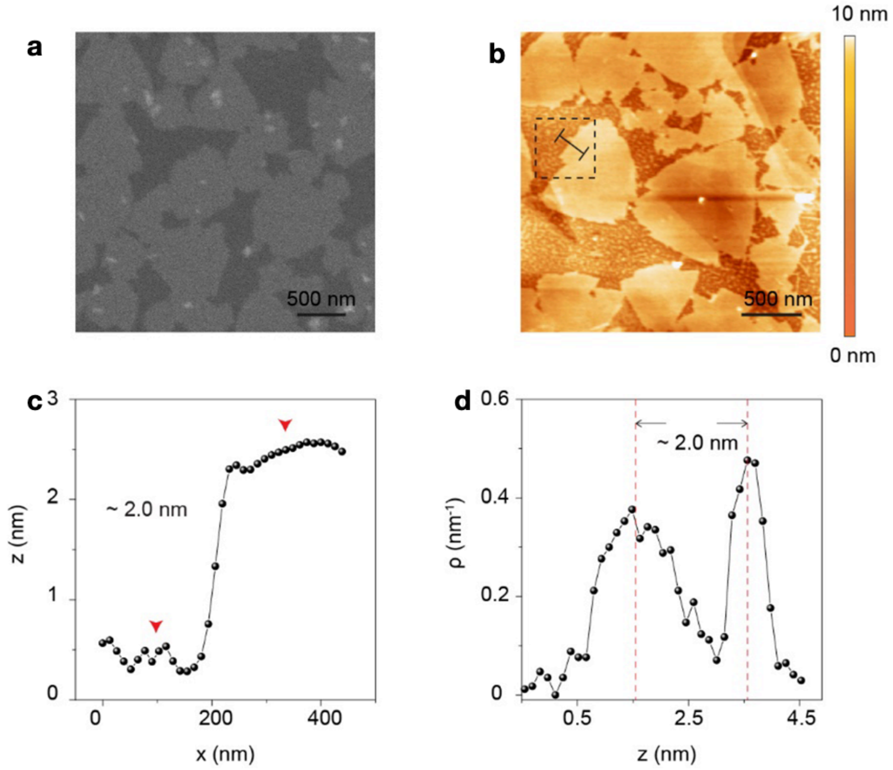

Supplementary Fig. 2. SEM (**a**) and AFM (**b**) images of a submonolayer 2DZIF films. Corresponding height profiles in (**c**) and height distribution in (**d**), acquired from line and square area labelled in (**b**). Reaction condition, 1 mM Zn^{2+}; 8 mM 2-mIm; reaction time of 2 min.

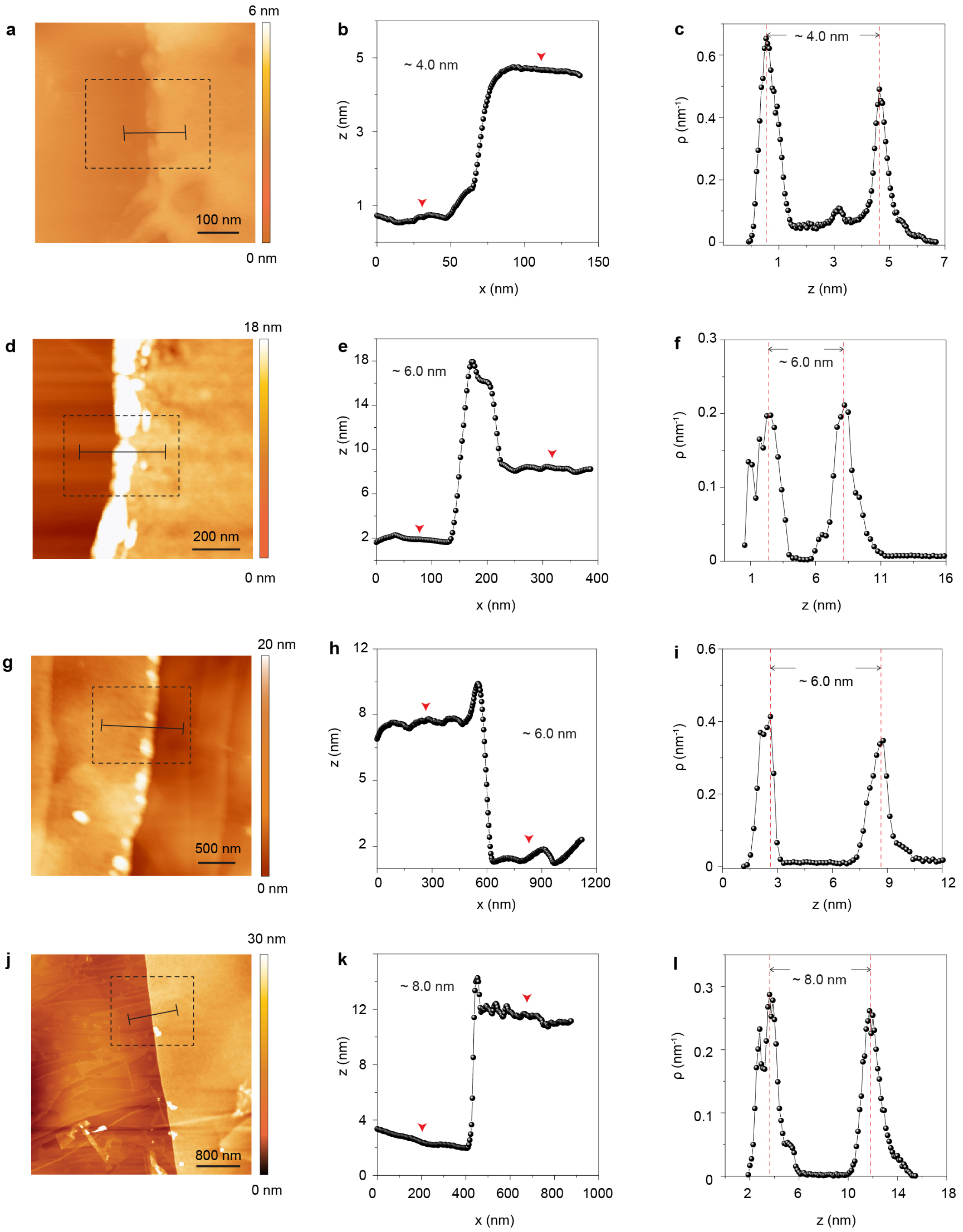

Supplementary Fig. 3. AFM images and the corresponding height profiles and height distributions for the 2DZIF films synthesized on HOPG with various thickness by changing synthesis time and precursor concentration (all height profiles and distributions were acquired from corresponding labelled lines and areas in the corresponding AFM images),

(**a**, **b** and **c**) 1 mM Zn^{2+}; 8 mM 2-mIm; reaction time of 10 min.

(**d**, **e** and **f**) 1 mM Zn^{2+}; 8 mM 2-mIm; reaction time of 15 min.

(**g**, **h** and **i**) 1 mM Zn^{2+}; 8 mM 2-mIm; reaction time of 20 min.

(**j**, **k** and **l**) 2 mM Zn^{2+}; 16 mM 2-mIm; reaction time of 2 min.

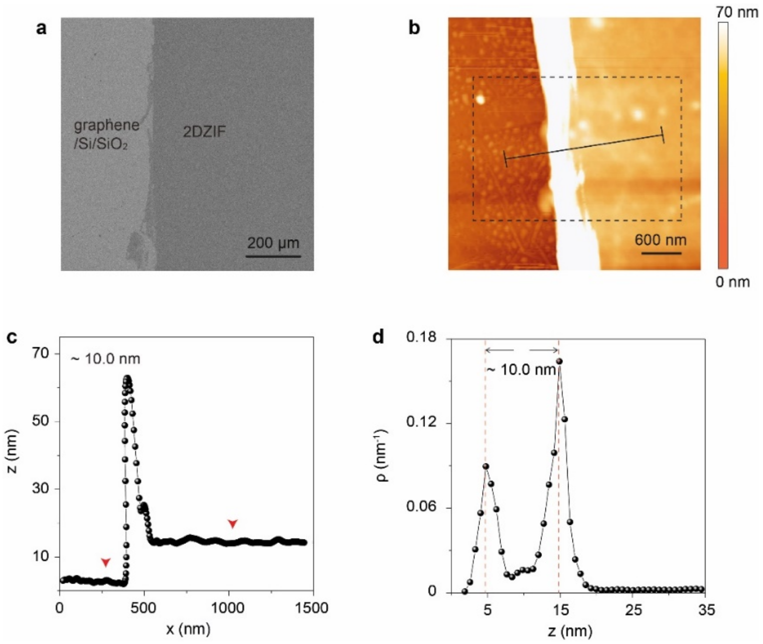

Supplementary Fig. 4. SEM (**a**) and AFM (**b**) images of a 2DZIF film on graphene/Si/SiO_2 used for the GIXRD measurement. (**c**) Height profile and (**d**) height distribution from AFM, acquired from line and square area labelled in (**b**).

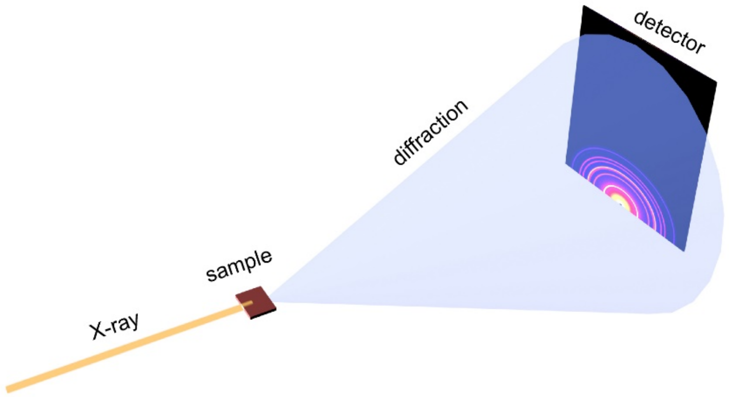

Supplementary Fig. 5. Schematic of GIXRD measurement.

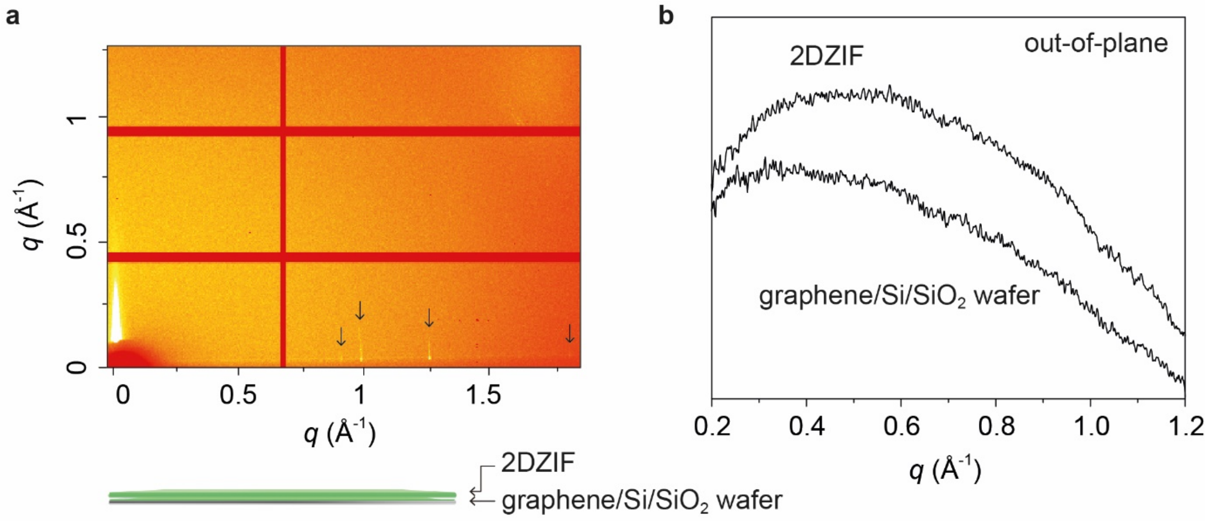

Supplementary Fig. 6. **a**, GIXRD pattern from 2DZIF film prepared on graphene/Si/SiO_2, arrows represent the diffractions. **b**, Integrated out-of-plane data. The area of 2DZIF involved in the diffraction is 200 μm × 5 mm. The measurement geometry of this sample was the same as that from 2DZIF@Sapphire (Supplementary Fig. 9g).

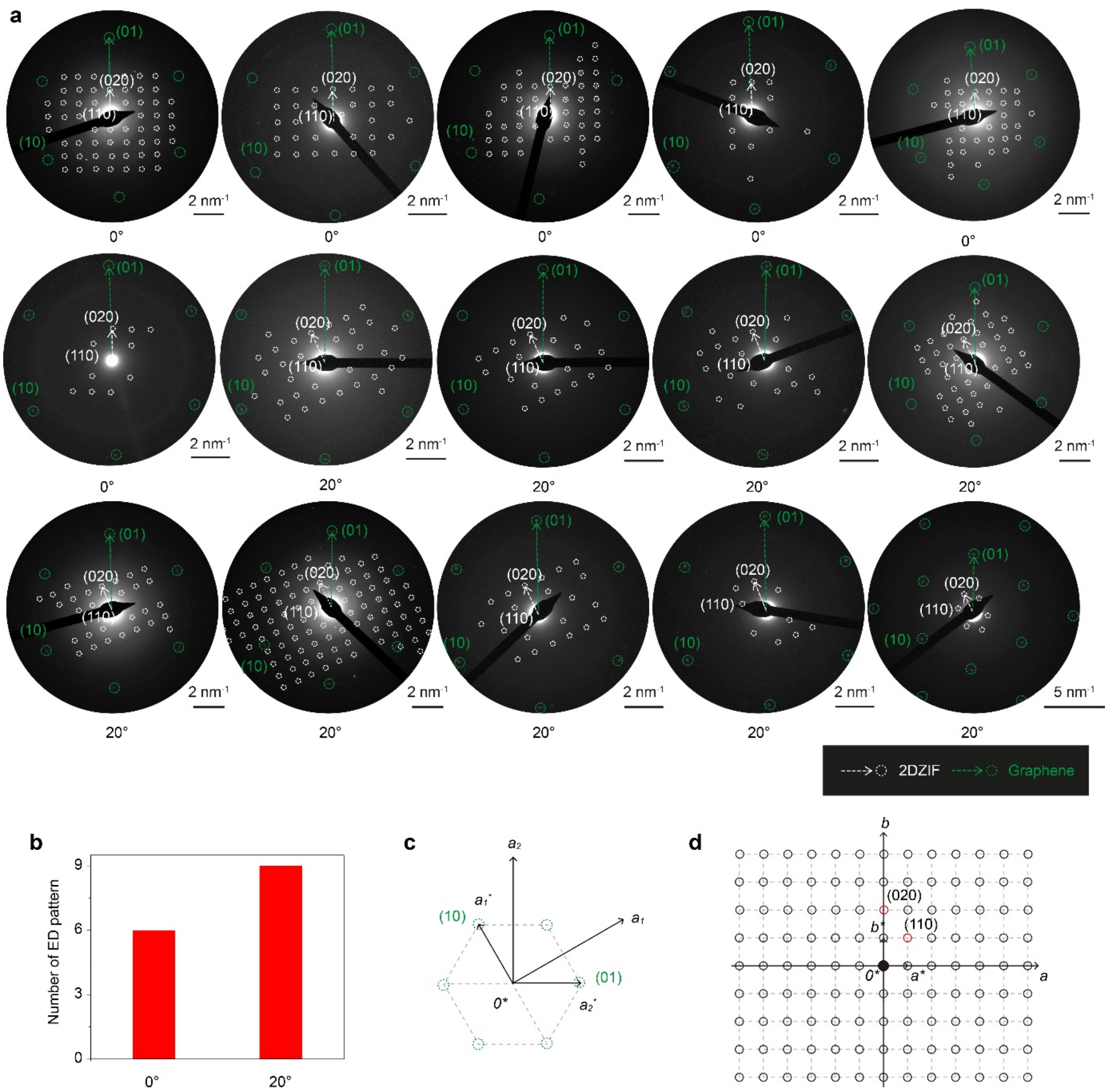

Supplementary Fig. 7. Analysis of orientation of 2DZIF on graphene from SAED measurement. **a**, the angle between the [01] direction of graphene and [010] direction of 2DZIF is mentioned below each ED. **b**, the corresponding histogram of the orientation distribution is strictly bimodal (0 and 20 º). **c**, illustration of reciprocal lattice of graphene. **d**, illustration of reciprocal lattice of 2DZIF, with zone axis of [001].

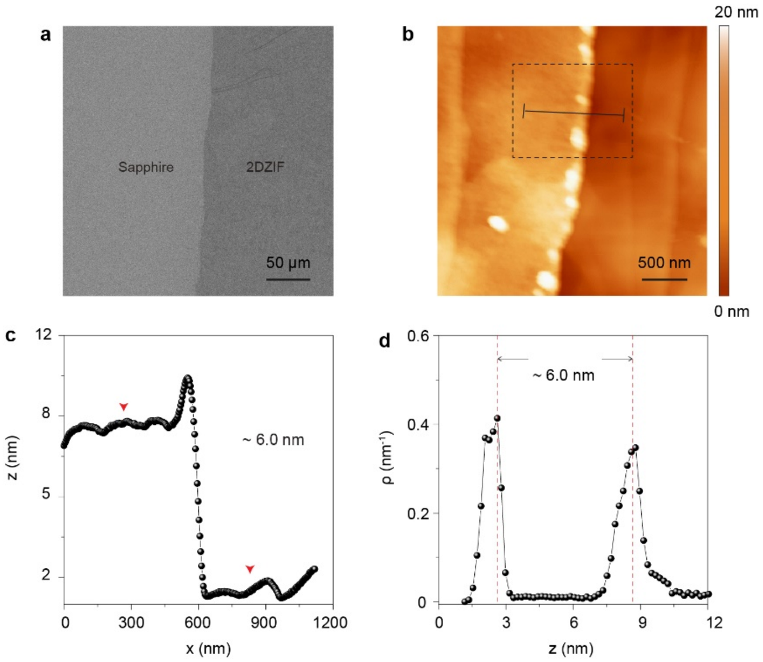

Supplementary Fig. 8. SEM (**a**) and AFM (**b**) images of a 2DZIF film formed on single crystal sapphire (Al_2O_3). (**c**) Height profile and (**d**) height distribution from AFM, acquired from line and square area labelled in (**b**). Reaction condition, 2 mM Zn^{2+}; 16 mM 2-mIm; reaction time of 2 min.

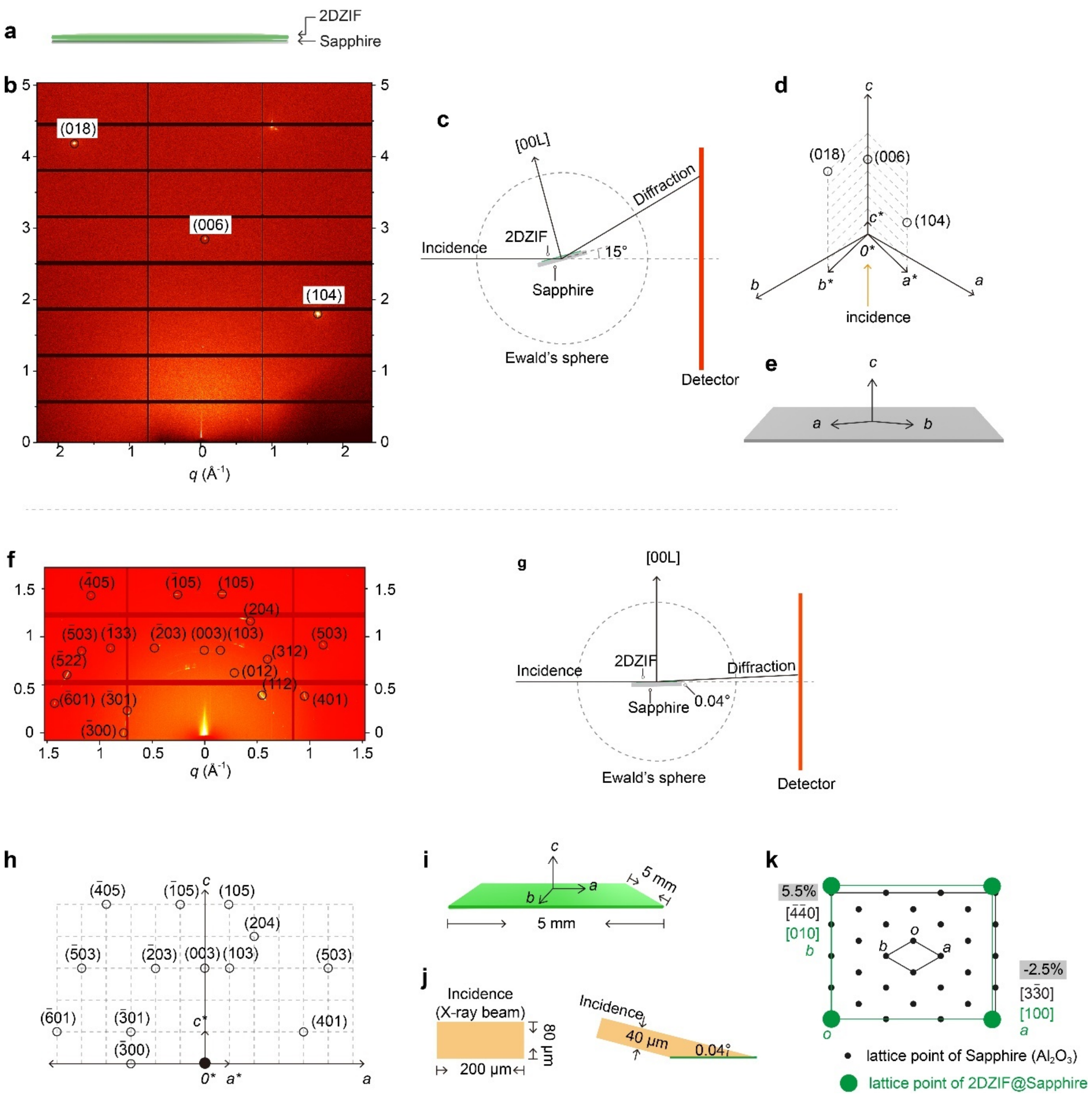

Supplementary Fig. 9. GIXRD pattern from 2DZIF film prepared on sapphire (Al_2O_3). **a**, schematic of the sample. **b**, and **c**, diffraction and corresponding illustration of measurement geometry of sapphire (Al_2O_3) from 2DZIF@sapphire sample. **d**, illustration of reciprocal lattice of sapphire. **e**, index of the lattice orientation of sapphire (Al_2O_3) substrate. **f** and **g**, diffraction and corresponding illustration of measurement geometry of 2DZIF from 2DZIF@sapphire sample. **h**, illustration of reciprocal lattice of sapphire, with zone axis of [001]. **i**, index of lattice orientation of 2DZIF and the size of sample was 5 × 5 mm. **j**, illustration of incident X-ray beam and its irradiation on sample. **k**, illustration of lattice mismatch calculation between 2DZIF and sapphire. Clear preferential orientation was proved by the dot-pattern from the GIXRD data of 2DZIF.

As for the index of lattice orientation of the sapphire, since it was rotated 15° of the measurement of sapphire, and was identified by the projection as shown in panel **c** and **d**, only when the incidence was located in between a* and b* lattice axis of sapphire, the diffraction in panel **b** can be observed, which evidenced the lattice orientation as showed in panel **e**. While for the the index of lattice orientation of the 2DZIF, the sample was almost horizontally positioned. It was identified by the projection as shown in panel **g** and **h**, where (101) lattice plane was parallel to the detector plane. It is worth noting that, most of these dots in panel **f** can be indexed from one single orientation, where only a few, ($\bar{5}22$), ($\bar{1}33$), (012) and (312), are not belonging to this grain, as showed in panel **h**. As shown in panel **j**, the shading area of X-ray is 200 μm × {40 μm/[sin(0.04°)]} = 200 μm × 57 mm. The size of sample was 5 mm × 5 mm (**l**), which means the area of 2DZIF involved in the diffraction is 200 μm × 5 mm.

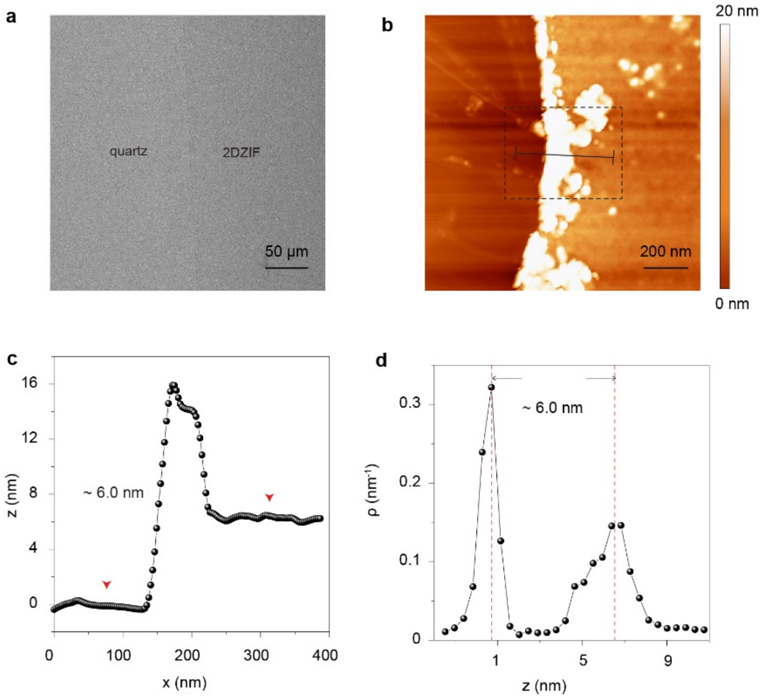

Supplementary Fig. 10. SEM (**a**) and AFM (**b**) images of a 2DZIF film on single-crystal quartz, that is used for the GIXRD measurement. (**c**) Height profile and (**d**) height distribution from AFM, acquired from line and square area labelled in (**b**). Reaction condition, 2 mM Zn^{2+}; 16 mM 2-mIm; reaction time of 2 min.

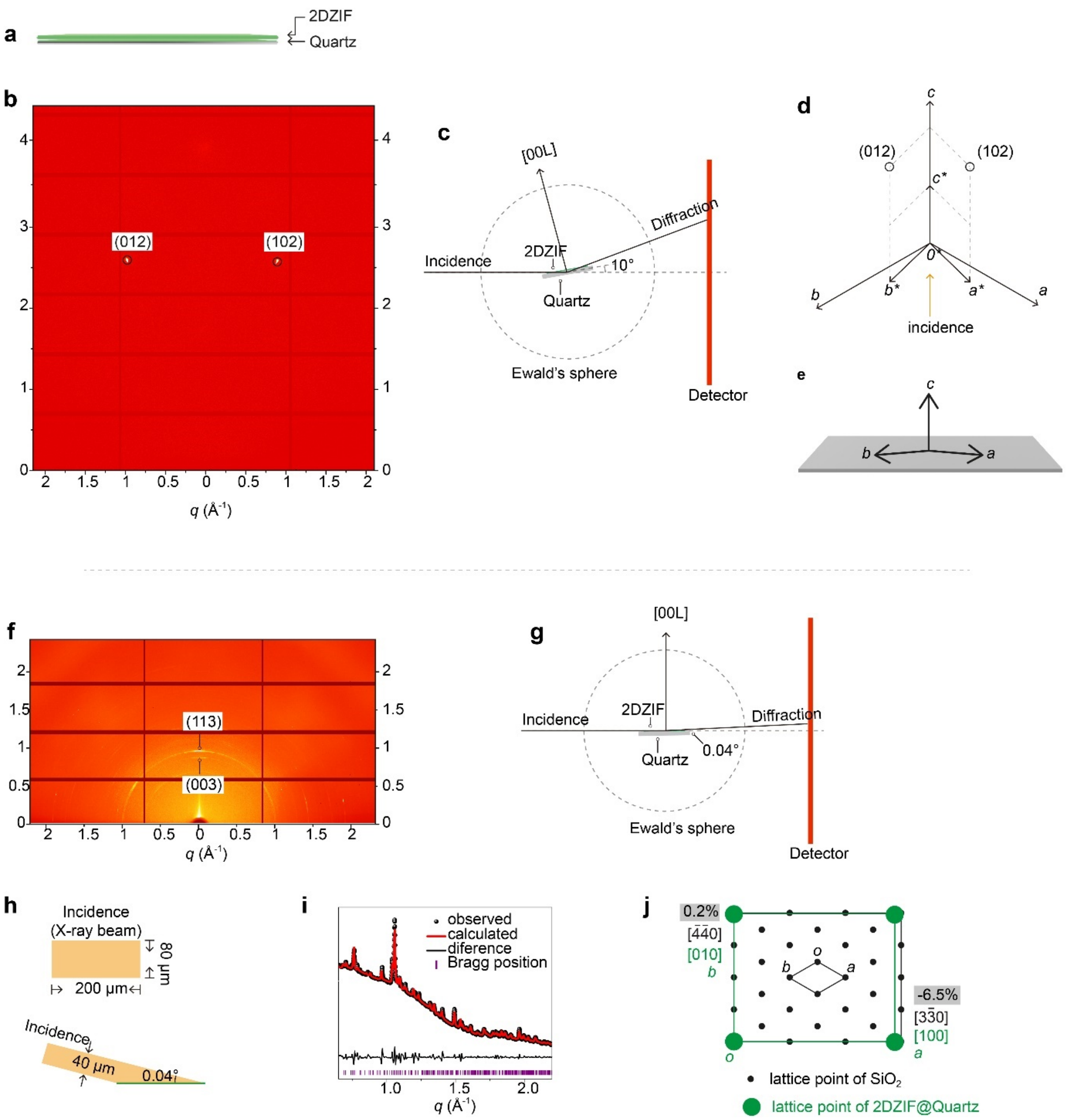

Supplementary Fig. 11. GIXRD pattern from 2DZIF film prepared on single-crystal quartz. **a**, schematic of the sample. **b**, and **c**, diffraction and corresponding illustration of measurement geometry of quartz (SiO_2) from 2DZIF@quartz sample. **d**, illustration of reciprocal lattice of quartz. **e**, index of the lattice orientation of quartz (SiO_2) substrate. **f** and **g**, diffraction and corresponding illustration of measurement geometry of 2DZIF from 2DZIF@quartz sample. **h**, illustration of incident X-ray beam and its irradiation on sample. **i**, Le-Bail fitting of integrated diffraction from all data of panel **f**. **j**, lattice mismatch calculation of 2DZIF and quartz. Clear preferential orientation was proved by the pattern from the GIXRD data of 2DZIF.

As for the index of lattice orientation of the quartz, since it was rotated 10° of the measurement of sapphire, and was identified by the projection as shown in panel **c** and **d**. Only when the incidence was located in between a* and b* lattice axis of quartz, the diffraction in panel **b** can be observed, which evidenced the lattice orientation as showed in panel **e**. While for the the index of lattice orientation of the 2DZIF, the sample was almost horizontally positioned. Since the in-plane orientation of 2DZIF film was not indexed, here [0k0] lattice axis from 2DZIF was assumed to be parallel to [hk0] lattice axis from SiO_2 (**j**), the same as that observed in 2DZIF@Sapphire sample (panel **k**, **Supplementary Fig. 9**). As shown in panel **h**, the shading area of X-ray is 200 μm × {40 μm/[sin(0.04°)]} = 200 μm × 57 mm. The size of sample was 1 cm × 1cm, which means the area of 2DZIF involved in the diffraction is 200 μm × 1 cm.

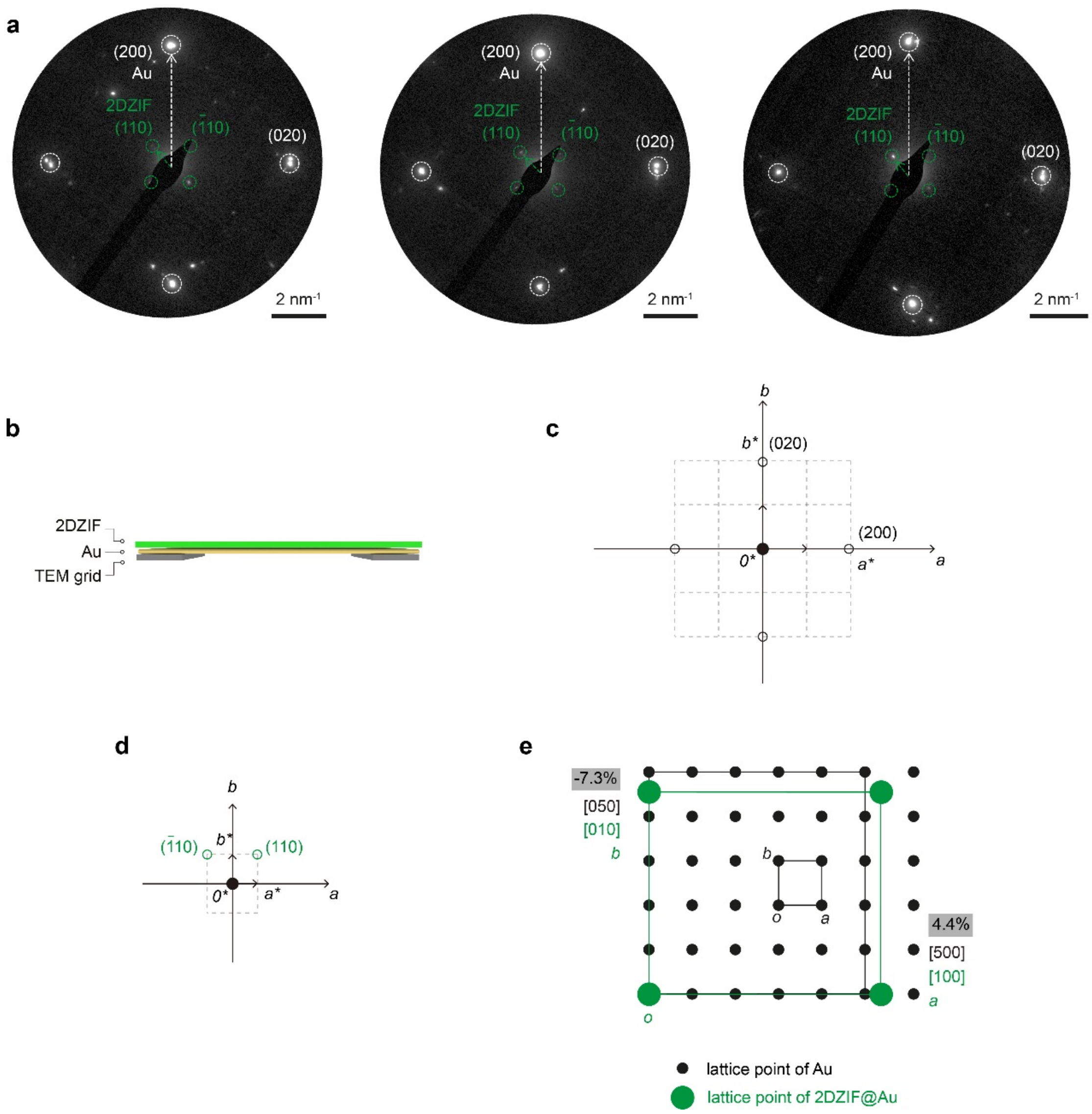

Supplementary Fig. 12. a, selected area electron diffraction (SAED) of 2DZIF grown on poly-crystalline Au thin film. **b**, corresponding schematic of the sample. **c**, illustration of reciprocal lattice of Au, with zone axis of [001]. **d**, illustration of reciprocal lattice of 2DZIF, with zone axis of [001]. **e**, illustration of lattice mismatch between 2DZIF and Au. Aperture size was 1 µm.

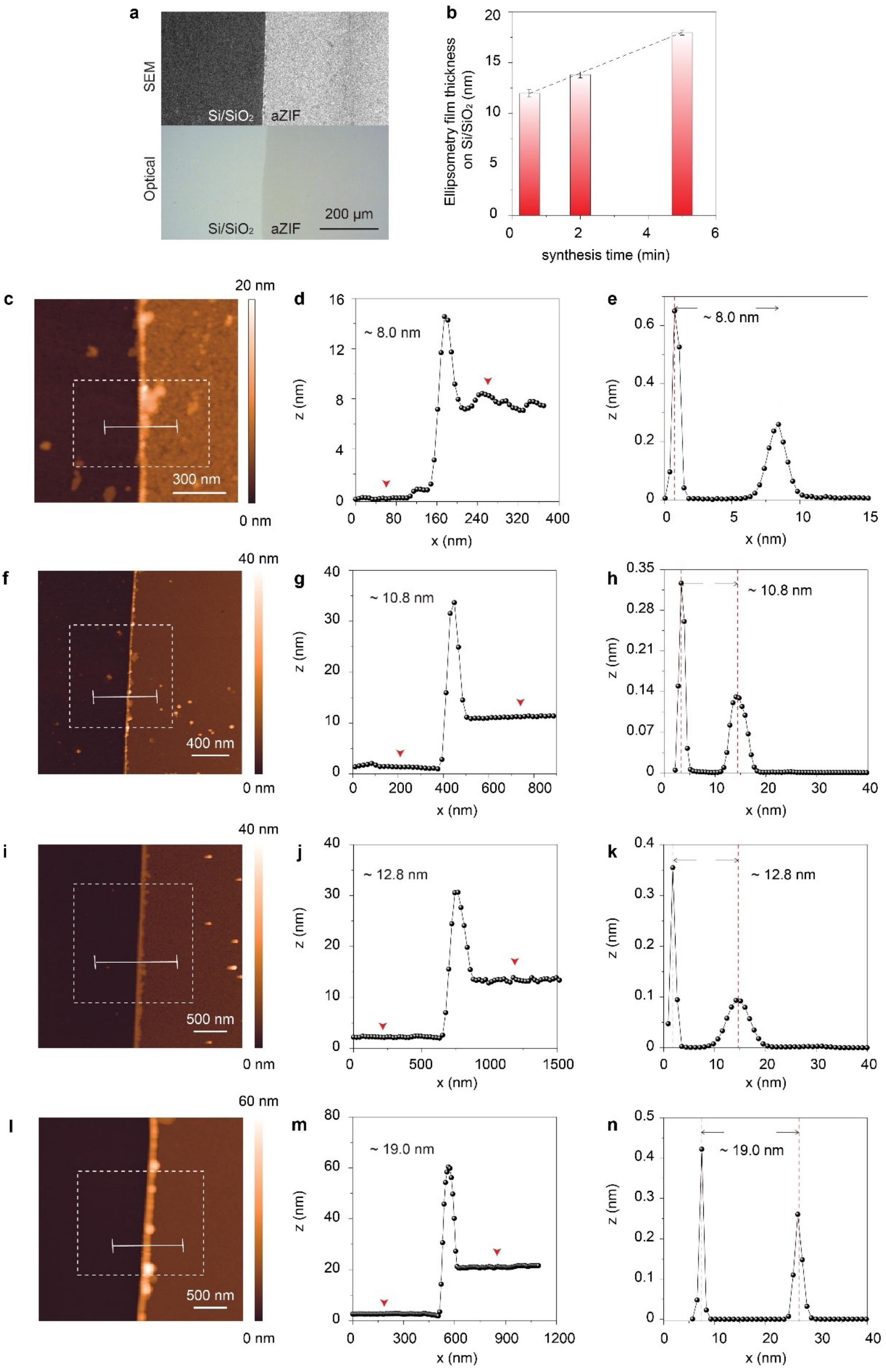

Supplementary Fig. 13. a, SEM and optical images of aZIF film on Si/SiO_2. **b**, Ellipsometry thickness of several ZIF films on a Si/SiO_2 wafer prepared using varying reaction times, error bars in this figure represent the standard deviation of difference in the thickness within 3

measurements and the center of each error bar represents the average thickness of the film., and **c**-**n**, AFM images and the corresponding height profiles and height distributions for aZIF films prepared on Si/SiO_2 wafer using 2 mM Zn^{2+} and 16 mM 2-mIm. All height profiles and distributions were acquired from corresponding labelled lines and areas in the corresponding AFM images. The reaction times were as follows:
(**c**, **d** and **e**) 10 s. (**f**, **g** and **h**) 30 s. (**i**, **j** and **k**) 2 min. (**l**, **m** and **n**) 5 min.

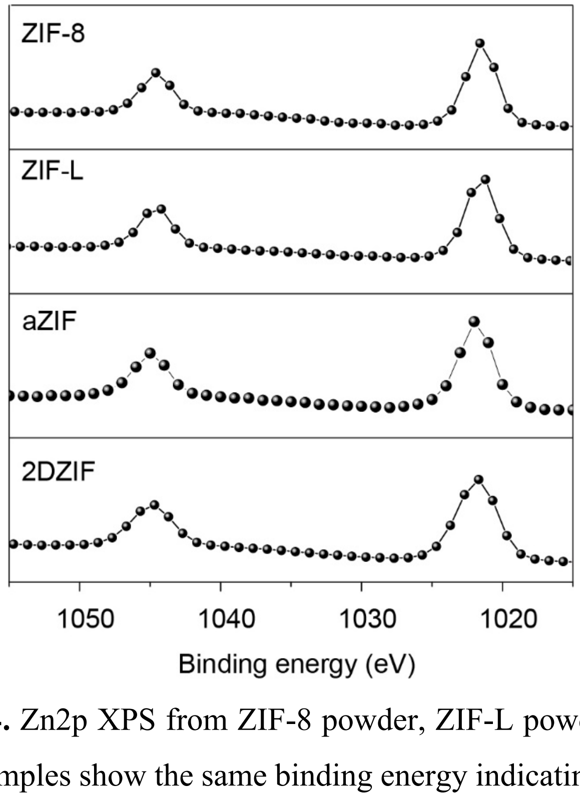

Supplementary Fig. 14. Zn2p XPS from ZIF-8 powder, ZIF-L powder compared with aZIF and 2DZIF films. All samples show the same binding energy indicating the same coordination environment of Zn in these samples.

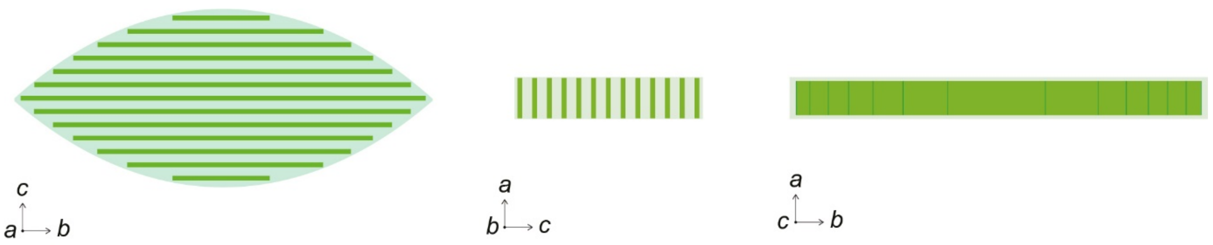

Supplementary Fig. 15. Schematic of the arrangement of layers within a ZIF-L crystal.

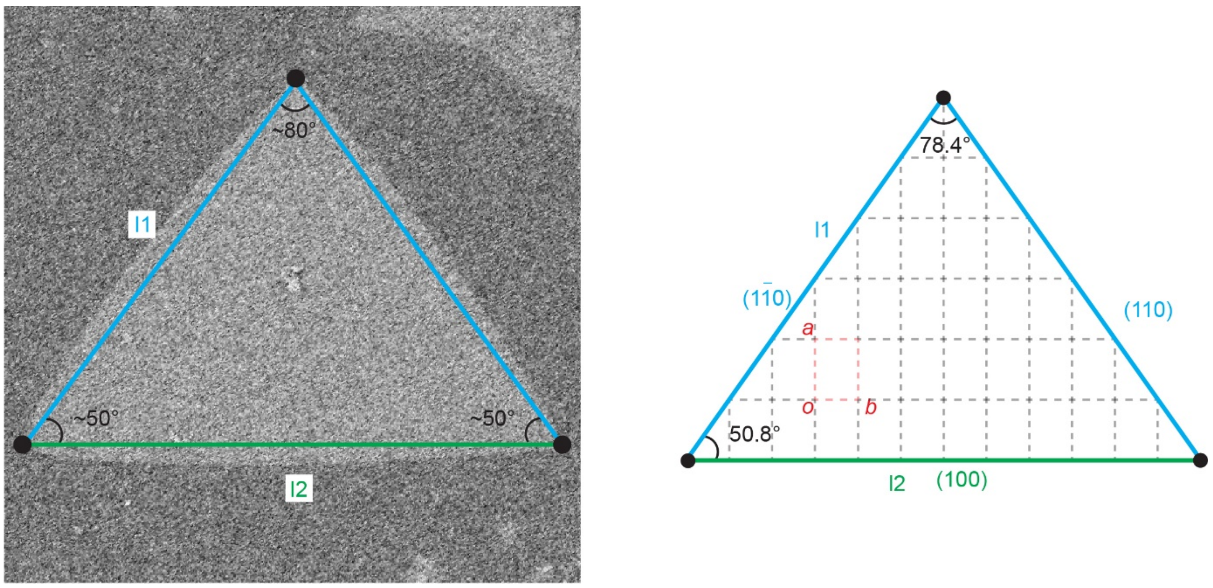

Supplementary Fig. 16. SEM image of a triangular 2DZIF grain (left) where the proposed lattice edges are highlighted and explained using a lattice model (right). In the model, the edge surface can be assigned to be (110), (1$\bar{1}$0) and (100) surfaces of 2DZIF, respectively, which constitute the lowest surface energies for 2D ZIF structures. For example, the reported surface energy of the (100) and (110) surfaces of ZIF-L are 0.106 J/m^2 and 0.116 J/m^2, respectively, much lower than that of the (010) surface (0.206 J/m^2) (reference 43 in main text).

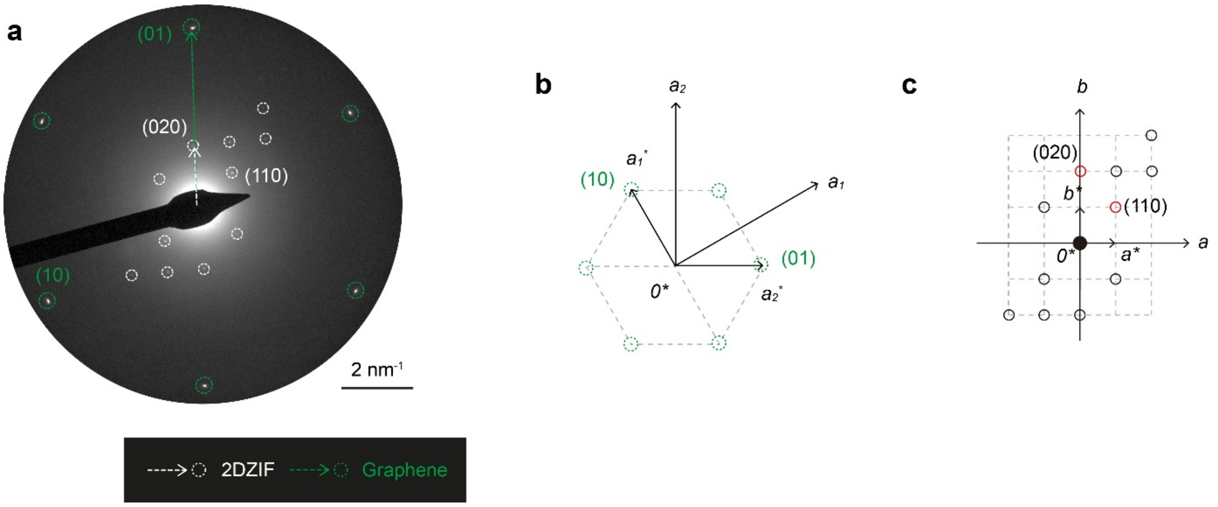

Supplementary Fig. 17. a, SAED of water etched (5 min) 2DZIF on graphene. **b**, illustration of reciprocal lattice of graphene. **c**, illustration of reciprocal lattice of 2DZIF, with zone axis of [001].

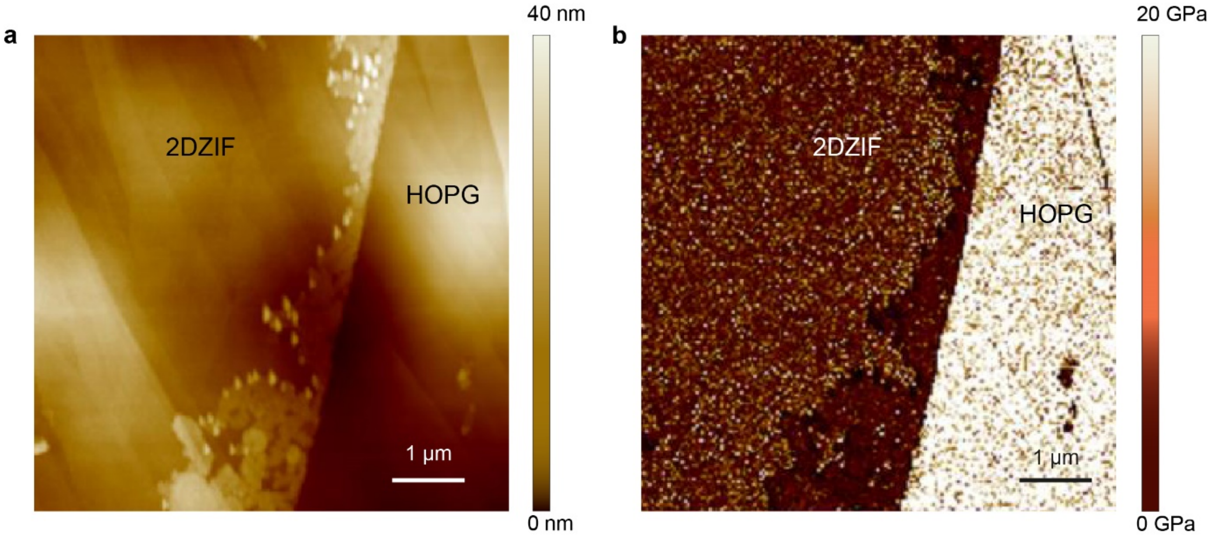

Supplementary Fig. 18. AFM image (**a**) and corresponding modulus map (**b**) of 2DZIF film synthesized on HOPG. The modulus is obtained by the PeakForce Tapping technique.

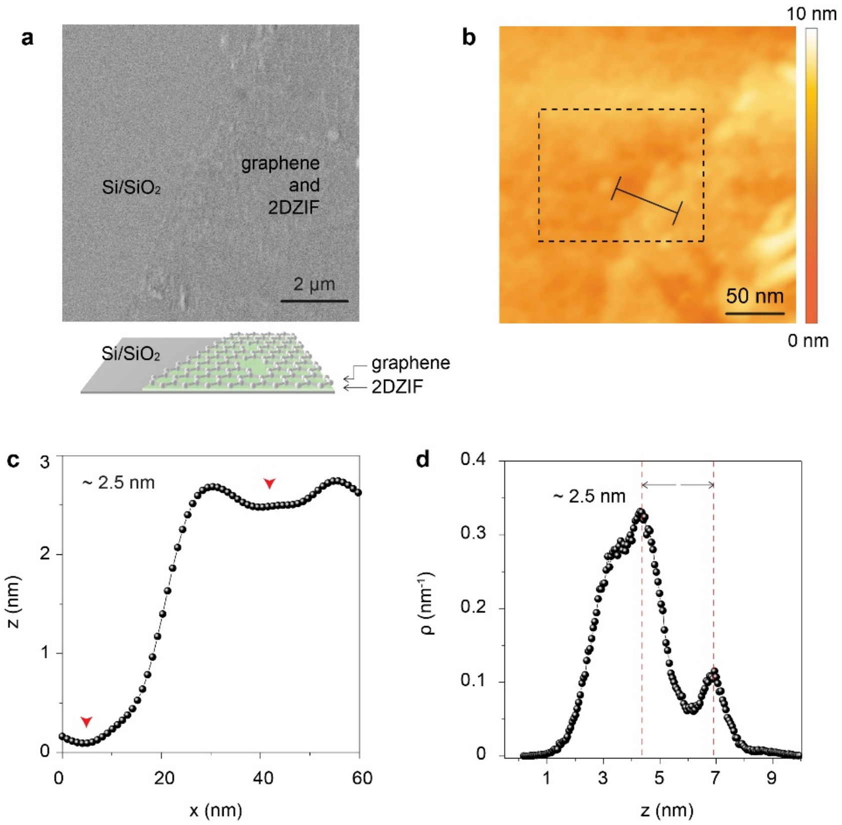

Supplementary Fig. 19. SEM (**a**) and AFM (**b**) images of 2DZIF/graphene membrane for gas separation. AFM height profile (**c**) and height distribution (**d**) corresponding to the line and rectangle in (**b**). The thickness of 2DZIF/graphene membrane is about 2.5 nm. Considering 0.3 nm thickness of single-layer graphene and van der Waals gap between 2DZIF and graphene, the thickness of 2DZIF layer is about 2.0 nm, consistent with the sample synthesized on HOPG.

Supplementary data for the gas separation performances of 2DZIF films

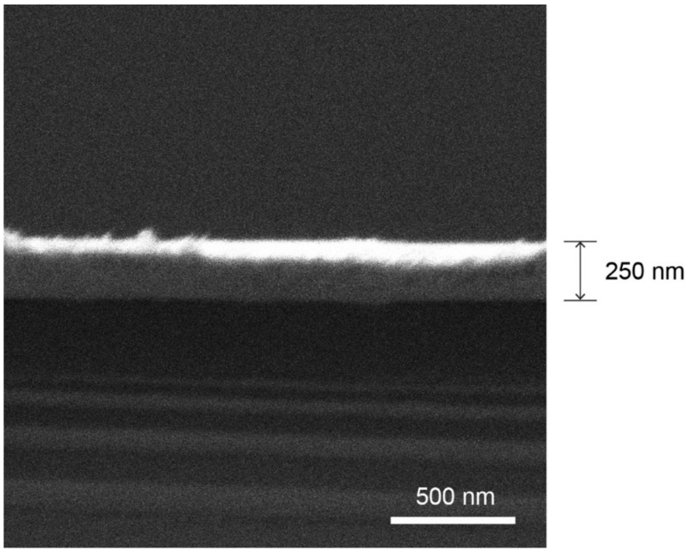

Supplementary Fig. 20. SEM image of the cross-section of the 2DZIF/graphene/PTMSP film on Si/SiO_2 wafer.

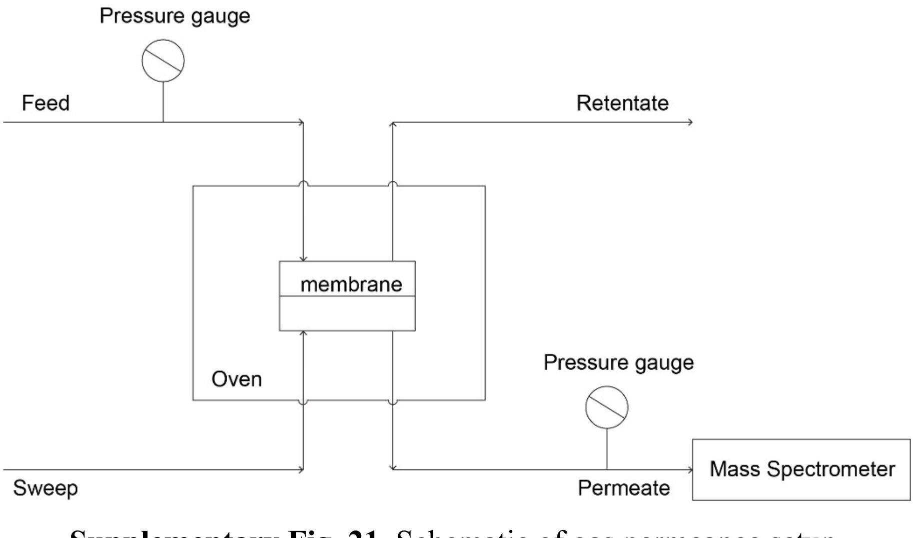

Supplementary Fig. 21. Schematic of gas permeance setup.

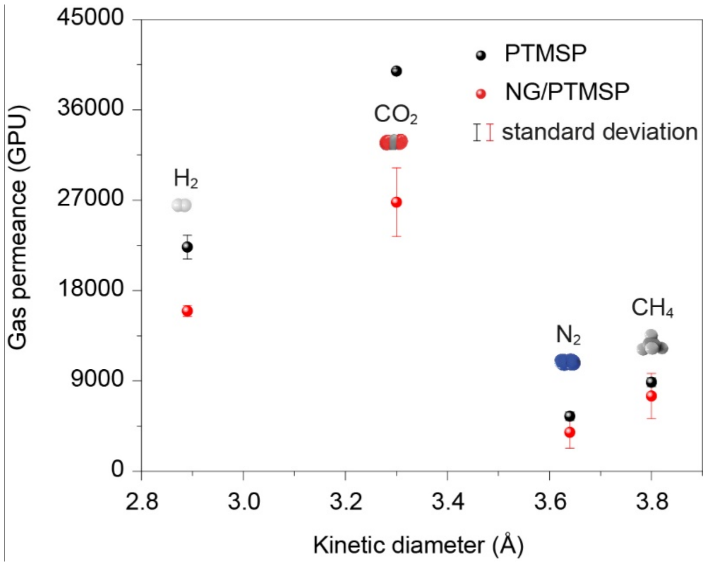

Supplementary Fig. 22. H_2, CO_2, N_2 and CH_4 permeances from the PTMSP and NG/PTMSP support films, where the error bar is the standard deviation of gas permeance of 3 batches of corresponding membranes and the center of each error bar represents the average gas permeance (Supplementary Table 7). The gas permeances are decreased by the addition of graphene to the support film while the gas pair selectivities are comparable, indicating nonselective nature of the NG layer used in this study.

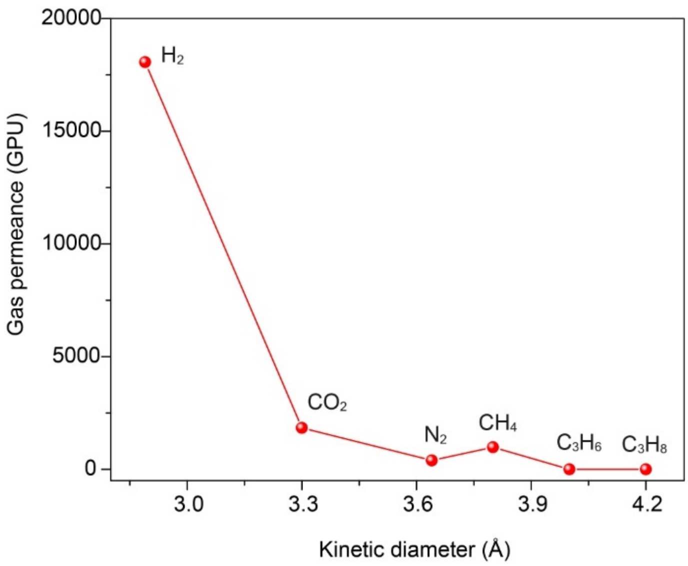

Supplementary Fig. 23. Single gas permeance of 2DZIF membrane.

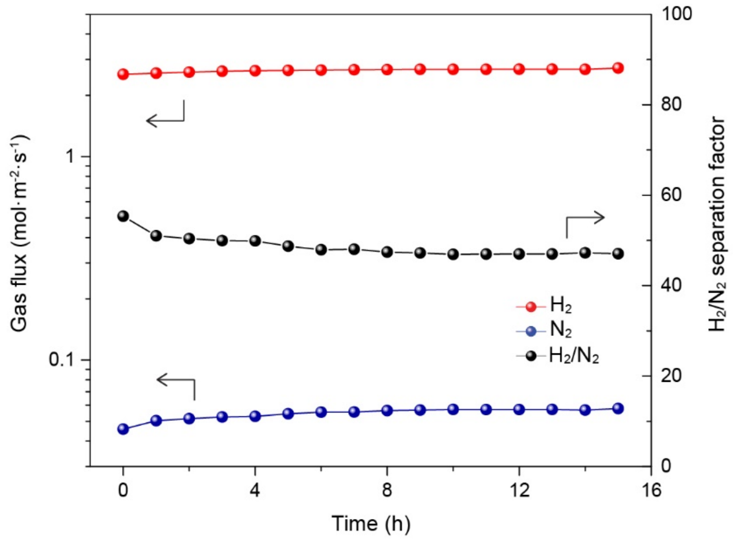

Supplementary Fig. 24. Separation performance from a 2DZIF membrane fed with an equimolar H_2/N_2 mixture at 8 bar.

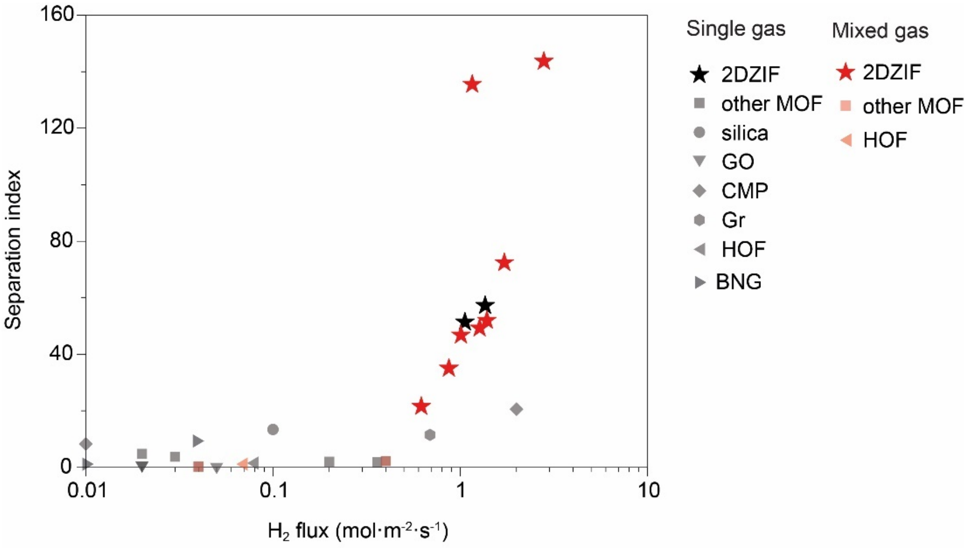

Supplementary Fig. 25. Separation index as a function of H_2 flux for 2DZIF membranes compared to other membranes in the literature. GO, CMP, HOF and BNG refer to graphene oxide, conjugated microporous polymers, hydrogen-bonded organic frameworks and boron nitride and graphene nanosheet, respectively.

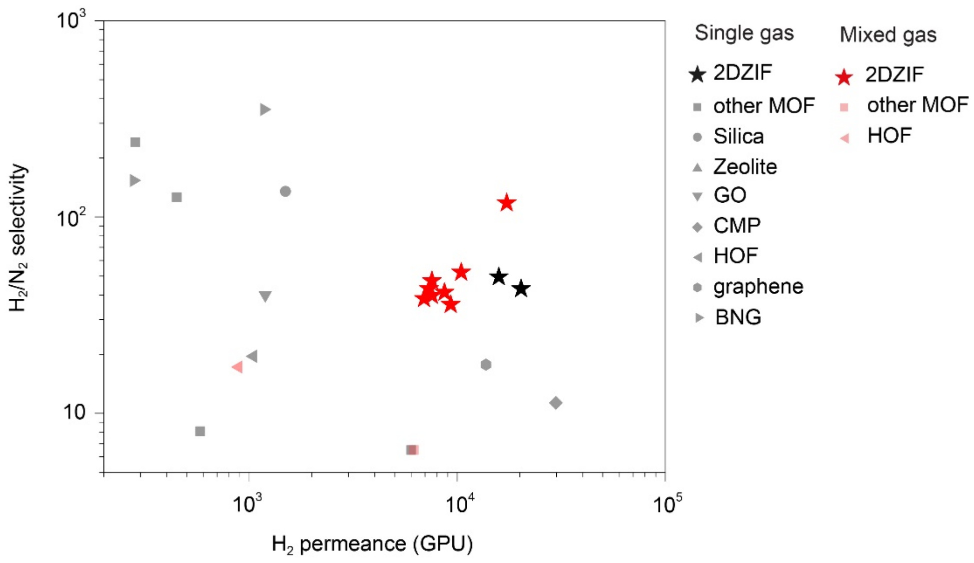

Supplementary Fig. 26. Comparison of the H_2/N_2 separation performance of 2DZIF membranes with the state-of-the-art. GO, CMP, HOF and BNG refer to graphene oxide, conjugated microporous polymers, hydrogen-bonded organic frameworks and boron nitride and graphene nanosheet, respectively.

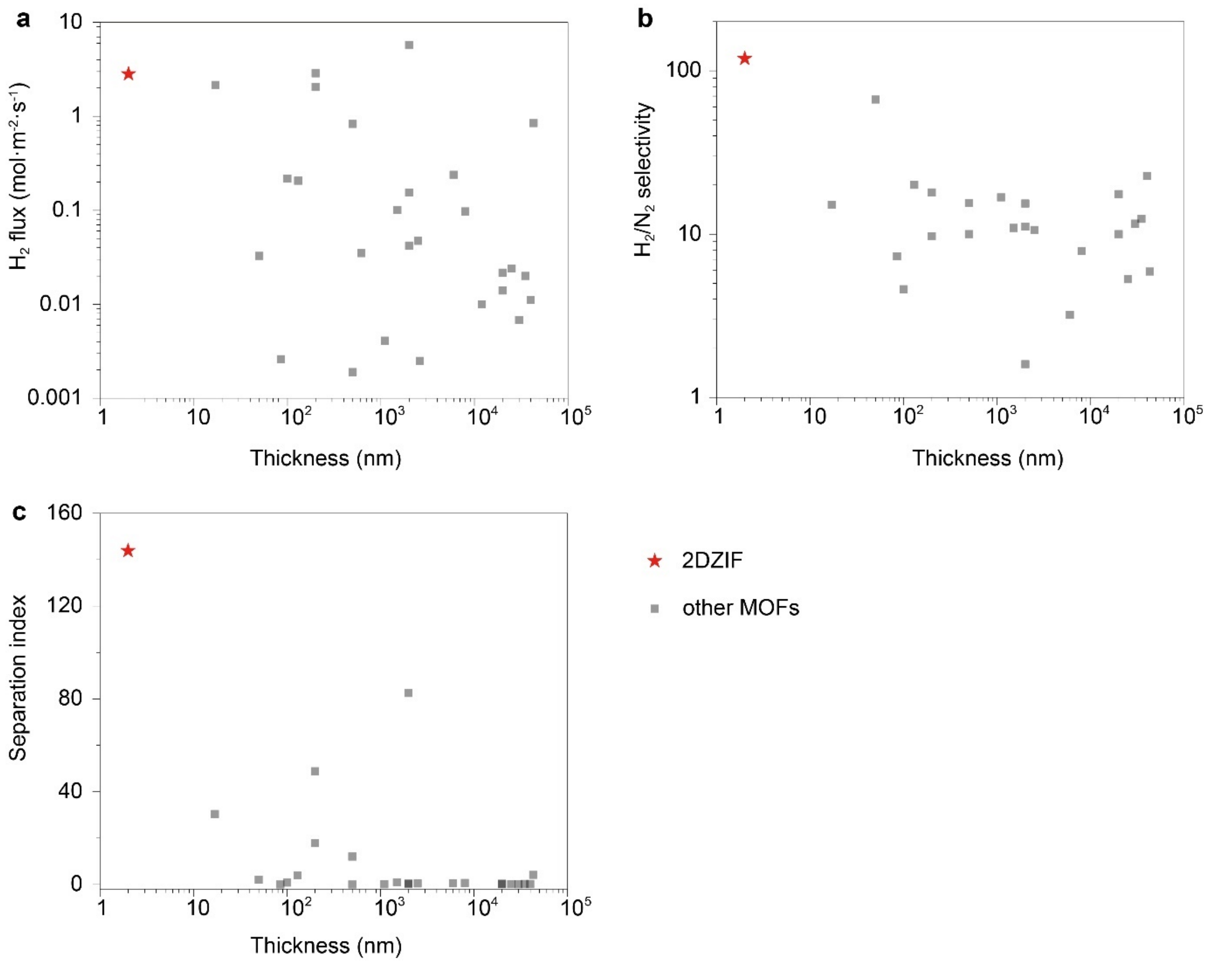

Supplementary Fig. 27. Comparisons of the H_2 flux (**a**), H_2/N_2 selectivity (**b**) and separation index (**c**) as a function of membrane thickness of 2DZIF membrane with other MOF membranes.

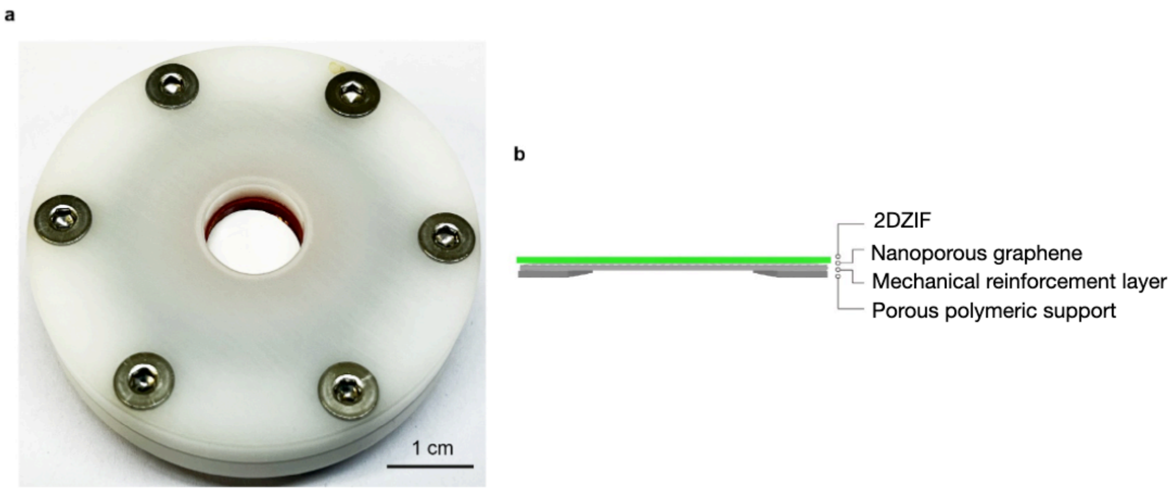

Supplementary Fig. 28. a, Optical picture of centimeter-scale 2DZIF membrane in a homemade membrane module. **b**, Schematic of assembly of the membrane inside the module. Nanoporous/2DZIF film was mechanically reinforced with a sub-micron Teflon AF film and then transferred on a porous polymeric support.

Supplementary data for e-beam patterning of aZIF films

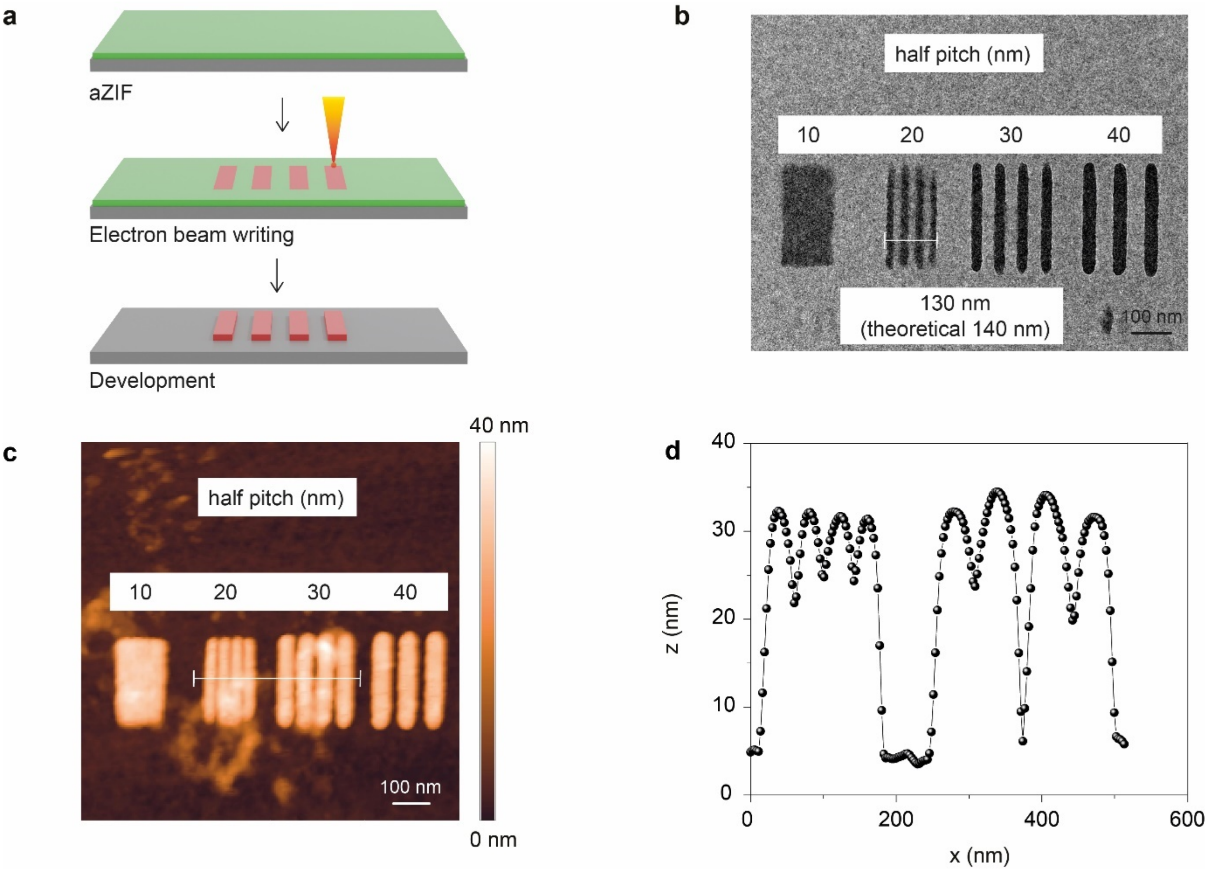

Supplementary Fig. 29. a, Schematic of the patterning process. TEM **(b)** and AFM **(c)** images of nanoscale patterns made on an aZIF film. **d,** AFM height profile corresponding to the line in **(c)**.

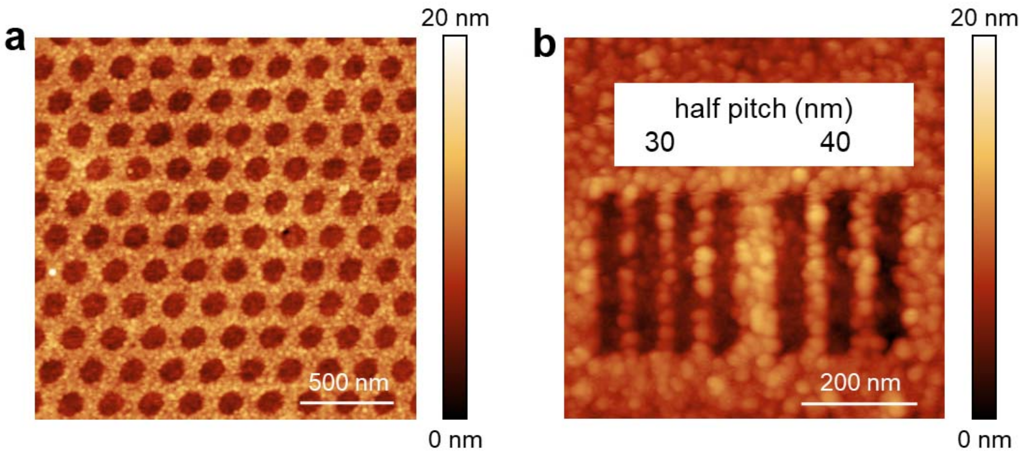

Supplementary Fig. 30. AFM images of (**a**) dot and (**b**) line patterns obtained from dcIm-treated aZIF in positive-tone patterning. Half pitches in (**b**) are indicated above the corresponding lines.

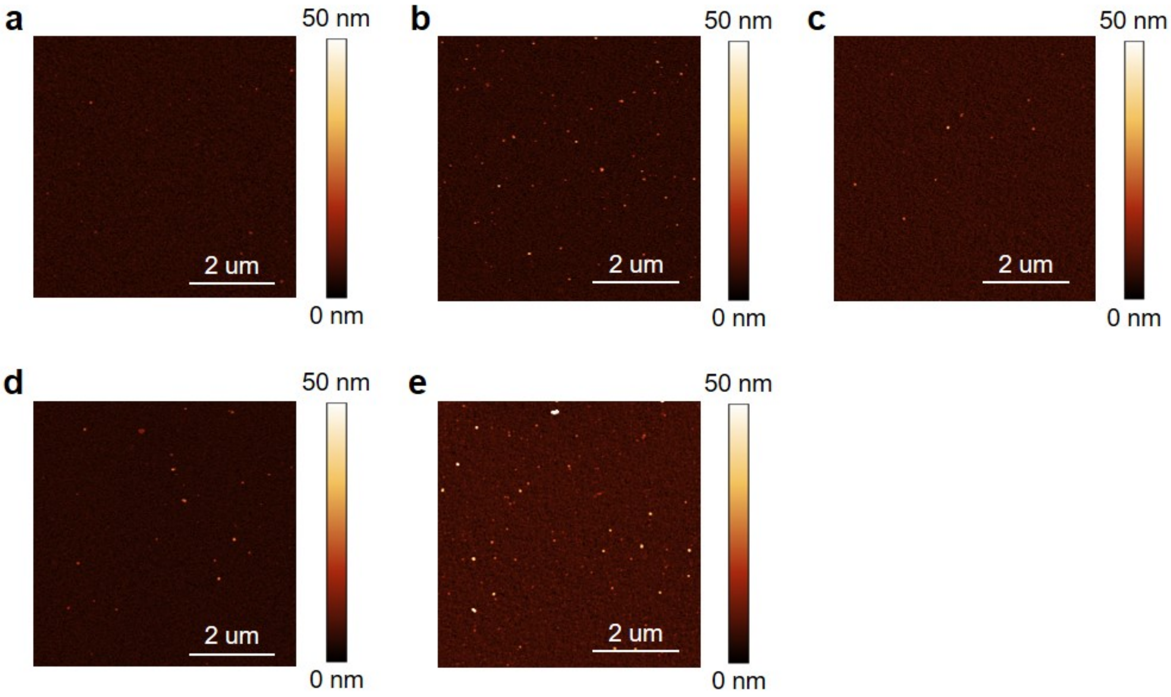

Supplementary Fig. 31. AFM images of aZIF films spin-coated at (**a**) 500, (**b**) 750, (**c**) 1000, (**d**) 1500 and (**e**) 2000 rpm, respectively.

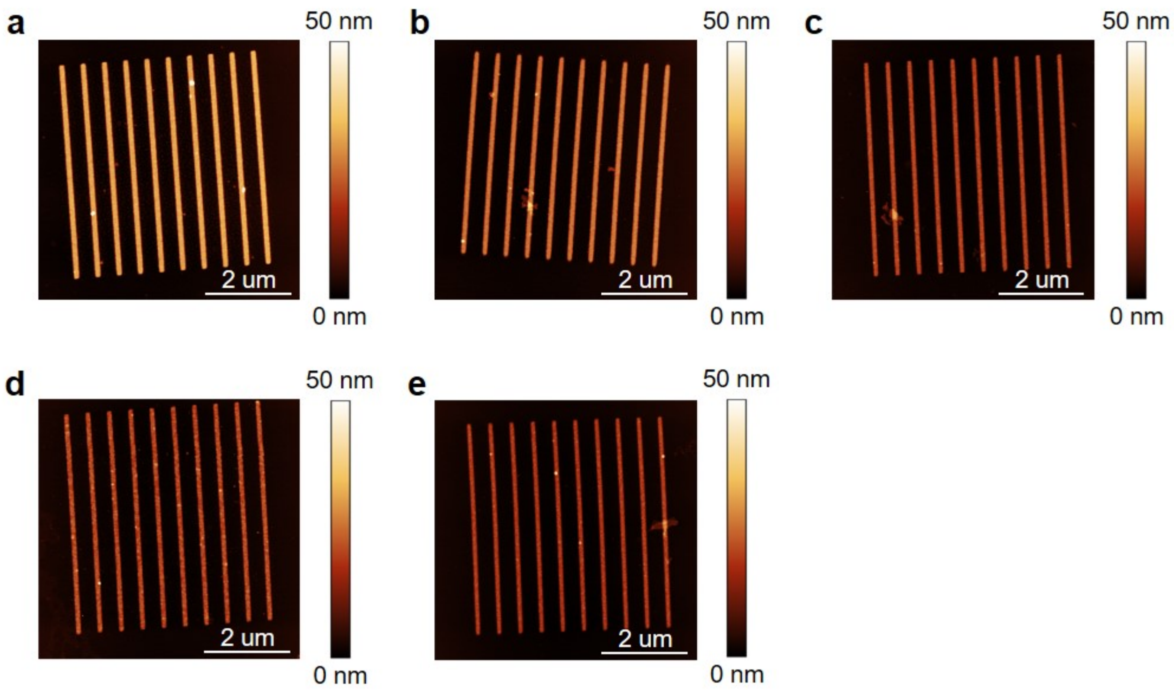

Supplementary Fig. 32. AFM images of line patterns with 100 nm width and 400 nm spacing after patterning on aZIF films spin-coated at (**a**) 500, (**b**) 750, (**c**) 1000, (**d**) 1500 and (**e**) 2000 rpm, respectively.

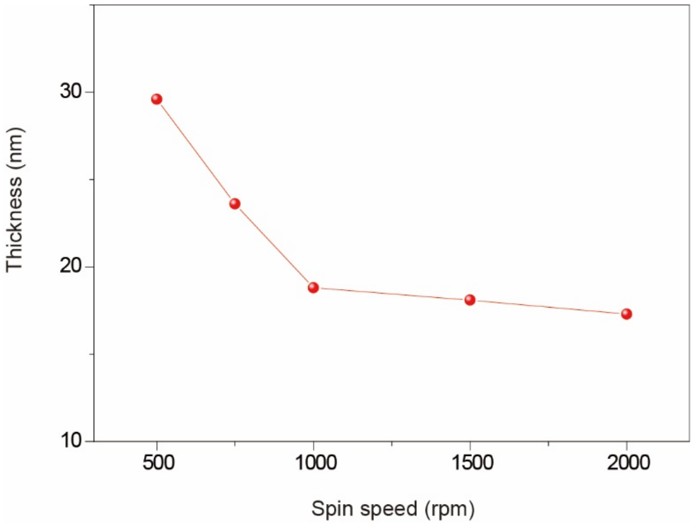

Supplementary Fig. 33. Thickness of aZIF films spin-coated at different speeds measured from height of line patterns in AFM.

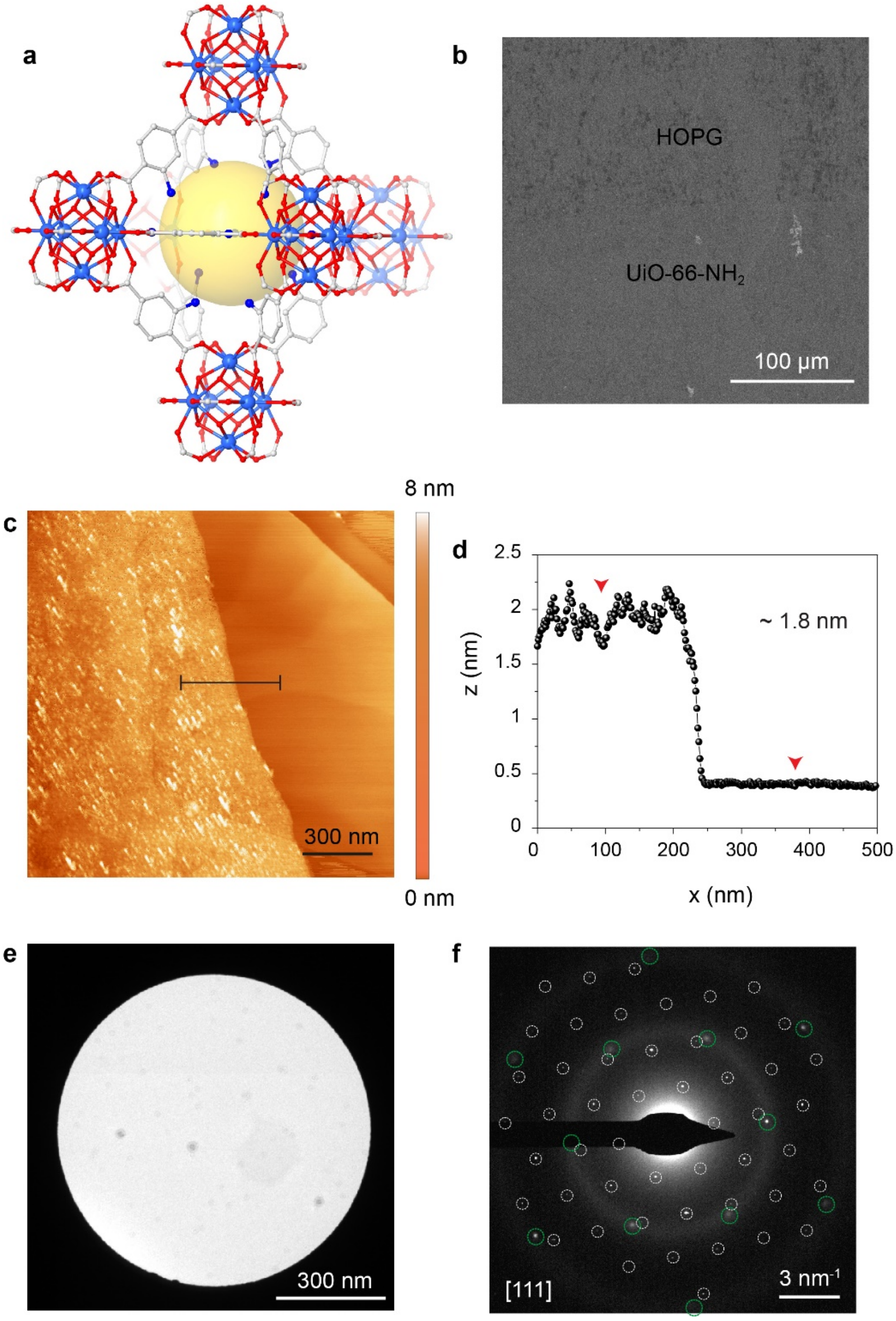

Supplementary Fig. 34. a, Structure of UiO-66-NH_2, **b**, SEM image of UiO-66-NH_2 film synthesized on HOPG. **c**, AFM image and corresponding height profile (**d**) for UiO-66-NH_2 film synthesized on HOPG, acquired from line labelled in (**c**). **e** and **f**, Bright-field TEM image of the UiO-66-NH_2 film supported on suspended graphene and its corresponding SAED pattern, where green and white circles are presenting diffraction from graphene and UiO-66-NH_2, respectively.

References

1. Dyadkin, V., Pattison, P., Dmitriev V., Chernyshov, D. A new multipurpose diffractometer PILATUS@SNBL. *J. Synchrotron Rad.* **23**, 825-829 (2016).

2. Bennett, T. D. & Horike, S. Liquid, glass and amorphous solid states of coordination polymers and metal–organic frameworks, *Nat. Rev. Mater.* **3**, 431-440 (2018).

3. Conrad, S. et al. Controlling Dissolution and Transformation of Zeolitic Imidazolate Frameworks by using Electron-Beam-Induced Amorphization, *Angew. Chem. Int. Ed.* **57**, 13592-13597 (2018).

4. Stowers, J. & Keszler, D. A. High resolution, high sensitivity inorganic resists, *Microelectron. Eng.* **86**, 730-733 (2009).

5. Miao, Y., Tsapatsis, M. Electron Beam Patterning of Metal–Organic Frameworks. *Chem. Mater.* **33**, 754-760 (2021).

6. Oleksak, R. P. et al. Chemical and Structural Investigation of High-Resolution Patterning with HafSOx, *ACS Appl. Mater. Interfaces* **6**, 2917-2921 (2014).

7. Stowers, J. & Keszler, D. A. High resolution, high sensitivity inorganic resists, *Microelectron. Eng.* **86**, 730-733 (2009).

8. Oleksak, R. P. et al. Chemical and Structural Investigation of High-Resolution Patterning with HafSOx, *ACS Appl. Mater. Interfaces* **6**, 2917-2921 (2014).

9. Ghosh, S. et al. *Chem. Mater.* Two distinct stages of structural modification of ZIF-L MOF under electron-beam irradiation. **33**, 5681-5689 (2021).

10. Luo, C. et al. Review of recent advances in inorganic photoresists, *RSC Adv.* **10**, 8385-8395 (2020).

11. Manouras, T. & Argitis, P. High Sensitivity Resists for EUV Lithography: A Review of Material Design Strategies and Performance Results, *Nanomaterials* **10**, 1593 (2020).

12. Gangnaik, A. S., Georgiev, Y. M. & Holmes, J. D. New Generation Electron Beam Resists: A Review, *Chem. Mater.* **29**, 1898-1917 (2017).

13. Ghash, S. et al. Two Distinct Stages of Structural Modification of ZIF-L MOF under Electron-Beam Irradiation, *Chem. Mater.* **33**, 5681-5689 (2021).

14. Wang, S. et al. Oriented Zeolitic Imidazolate Framework (ZIF) Nanocrystal Films for Molecular Separation Membranes. *ACS Appl. Nano Mater.* **3**, 3839−3846 (2020).

15. Kim, S. et al. The enhanced hydrogen separation performance of mixed matrix membranes by incorporation of two-dimensional ZIF-L into polyimide containing hydroxyl group. *J. Membr. Sci.* **549**, 260–266 (2018).

16. Zhang, J. et al. N-Doped hierarchically porous carbon derived from heterogeneous core–shell ZIF-L(Zn)@ZIF-67 for supercapacitor application. *New J. Chem.* **42**, 6719—6726 (2018).

17. Wang, S., Zang, B., Chang, Y. & Chen, H. Synthesis and carbon dioxide capture properties of flower-shaped zeolitic imidazolate framework-L. *CrystEngComm* **21**, 6536–6544 (2019).

18. Han, S. et al. Root-like polyamide membranes with fast water transport for high-performance nanofiltration. *J. Mater. Chem. A* **8**, 25028–25034 (2020).

19. Zhu, W., Li, X., Sun, Y., Guo, R. & Ding, S. Introducing hydrophilic ultra-thin ZIF-L into mixed matrix membranes for CO_2/CH_4 separation. *RSC Adv.* **9**, 23390-23399 (2019).

20. Yuan, H. et al. ZnO Nanosheets Abundant in Oxygen Vacancies Derived from Metal-Organic Frameworks for ppb-Level Gas Sensing. *Adv. Mater.* **31**, 1807161 (2019).

21. Pan, Y., Liu, Y., Zeng, G., Zhao, L. & Lai, Z. Rapid synthesis of zeolitic imidazolate framework-8 (ZIF-8) nanocrystals in an aqueous system. *Chem. Commun.* **47**, 2071-2073 (2011).

22. Huang, K., Wang, B., Chi, Y. & Li, K. High Propylene Selective Metal-Organic Framework Membranes Prepared in Confined Spaces via Convective Circulation Synthesis. *Adv. Mater. Interfaces* **5**, 1800287 (2018).

23. Li, W. et al. Transformation of metal-organic frameworks for molecular sieving membranes. *Nat. Commun.* **7**, 11315 (2016).

24. Zhong, Z. et al. Carbon composite membrane derived from a two-dimensional zeolitic imidazolate framework and its gas separation properties. *Carbon* **72**, 242-249 (2014).

25. Eum, K. et al. ZIF-8 Membrane Separation Performance Tuning by Vapor Phase Ligand Treatment. *Angew. Chemie. Int. Ed.* **131**, 16542-16546 (2019).

26. De Vos, R. M. & Verweij, H. High-Selectivity, High-Flux Silica Membranes for Gas Separation. *Science* **279**, 1710-1711 (1998).

27. Poshusta, J. C., Tuan, V. A., Falconer, J. L. & Noble, R. D. Synthesis and Permeation Properties of SAPO-34 Tubular Membranes. *Ind. Eng. Chem. Res.* **37**, 3924-3929 (1998).

28. Guo, H. et al. Cross-Linking between Sodalite Nanoparticles and Graphene Oxide in Composite Membranes to Trigger High Gas Permeance, Selectivity, and Stability in Hydrogen Separation. *Angew. Chemie. Int. Ed.* **59**, 6284-6347 (2020).

29. Liu, W. et al. A solution-processable and ultra-permeable conjugated microporous thermoset for selective hydrogen separation. *Nat. Commun.* **11**, 1633 (2020).

30. Feng, S. et al. Fabrication of a Hydrogen-Bonded Organic Framework Membrane through Solution Processing for Pressure-Regulated Gas Separation. *Angew. Chemie. Int. Ed.* **59**, 3840-3845 (2020).

31. Wang, R. et al. Pyro-layered heterostructured nanosheet membrane for hydrogen separation. *Nat. Commun.* **14**, 2161 (2023).

32. Xu, G. et al. Preparation of ZIF-8 membranes supported on ceramic hollow fibers from a concentrated synthesis gel. *J. Membr. Sci.* **385-386**, 187-193 (2011).

33. Pan, Y., Wang, B., & Lai, Z. Synthesis of ceramic hollow fiber supported zeolitic imidazolate framework-8 (ZIF-8) membranes with high hydrogen permeability. *J. Membr. Sci.* **421-422**, 292-298 (2012).

34. Liu, D. et al. Gas transport properties and propylene/propane separation characteristics of ZIF-8 membranes. *J. Membr. Sci.* **451**, 85-93 (2014).

35. Cacho-Bailo, F. et al. ZIF-8 continuous membrane on porous polysulfone for hydrogen separation. *J. Membr. Sci.* **464**, 119-126 (2014).

36. Shah, M. et al. One step in situ synthesis of supported zeolitic imidazolate framework ZIF-8 membranes: Role of sodium formate. *Microporous Mesoporous Mater.* **165**, 63-69 (2013).

37. Barankova, E. et al. A Metal Chelating Porous Polymeric Support: The Missing Link for a Defect-Free Metal-Organic Framework Composite Membrane. *Angew. Chem. Int. Ed.* **56**, 2965-2968 (2017).

38. Liu, Y. et al. Remarkably Enhanced Gas Separation by Partial Self-Conversion of a Laminated Membrane to Metal-Organic Frameworks. *Angew. Chem. Int. Ed.* **54**, 3028-3032 (2015).

39. Xie, Z. et al. Deposition of chemically modified α-Al2O3 particles for high performance ZIF-8 membrane on a macroporous tube. *Chem. Commun.* **48**, 5977-5979 (2012).

40. Huang, K. et al. Growth of a ZIF-8 membrane on the inner-surface of a ceramic hollow fiber via cycling precursors. *Chem. Commun.* **49**, 10326-10328 (2013).

41. Shekhah, O. et al. The liquid phase epitaxy approach for the successful construction of ultra-thin and defect-free ZIF-8 membranes: pure and mixed gas transport study. *Chem. Commun.* **50**, 2089-2092 (2014).

42. Zhang, X. et al. A simple and scalable method for preparing low-defect ZIF-8 tubular membranes. *J. Mater. Chem. A* **1**, 10635-10638 (2013).

43. Shamsaei, E. et al. Rapid synthesis of ultrathin, defect-free ZIF-8 membranes via chemical vapour modification of a polymeric support. *Chem. Commun.* **51**, 11474-11477 (2015).

44. Cacho-Bailo, F. et al. MOF–polymer enhanced compatibility: post-annealed zeolite imidazolate framework membranes inside polyimide hollow fibers. *RSC Adv.* **6**, 5881-5889 (2016).

45. Su, P. et al. Metal based gels as versatile precursors to synthesize stiff and integrated MOF/polymer composite membranes. *J. Mater. Chem. A* **3**, 20345-20351 (2015).

46. Li, W. et al. Sol–gel asynchronous crystallization of ultra-selective metal-organic framework membranes for gas separation. *J. Mater. Chem. A* **6**, 16333-16340 (2018).

47. Shamsaei, E. et al. A one-dimensional material as a nano-scaffold and a pseudo-seed for facilitated growth of ultrathin, mechanically reinforced molecular sieving membranes. *Chem. Commun.* **52**, 13764-13767 (2016).

48. Li, W. et al. Ultrathin metal-organic framework membrane production by gel–vapour deposition. *Nat. Commun.* **8**, 406 (2017).

49. Bux, H. et al. Oriented Zeolitic Imidazolate Framework-8 Membrane with Sharp H_2/C_3H_8 Molecular Sieve Separation. *Chem. Mater.* **23**, 2262-2269 (2011).

50. Liu, Y. et al. *In Situ* Synthesis of MOF Membranes on ZnAl-CO_3 LDH Buffer Layer-Modified Substrates. *J. Am. Chem. Soc.* **136**, 14353-14356 (2014).

51. Bux, H. et al. Zeolitic Imidazolate Framework Membrane with Molecular Sieving Properties by Microwave-Assisted Solvothermal Synthesis. *J. Am. Chem. Soc.* **131**, 16000-16001 (2009).

52. Liu, Q. et al. Bio-Inspired Polydopamine: A Versatile and Powerful Platform for Covalent Synthesis of Molecular Sieve Membranes. *J. Am. Chem. Soc.* **135**, 17679-17682 (2013).

53. Li, W. et al. Metal-Organic Framework/PVDF Composite Membranes with High H_2 Permselectivity Synthesized by Ammoniation. *Chem. Eur. J.* **21**, 7224-7230 (2015).

54. Li, Y. et al. Growth of ZnO self-converted 2D nanosheet zeolitic imidazolate framework membranes by an ammonia-assisted strategy. *Nano Res.* **11**, 1850-1860 (2018).

55. Hu, Y. et al. Zeolitic Imidazolate Framework/Graphene Oxide Hybrid Nanosheets as Seeds for the Growth of Ultrathin Molecular Sieving Membranes. *Angew. Chem. Int. Ed.* **55**, 2048-2052 (2016).

56. Zhou, S. et al. Electrochemical synthesis of continuous metal-organic framework membranes for separation of hydrocarbons. *Nat. Energy* **6**, 882-891 (2021).

57. Fairley, K. C. et al. Sub-30 keV patterning of HafSOx resist: Effects of voltage on resolution, contrast, and sensitivity. *J. Vac. Sci. Technol. B* **34**, 041607 (2016).

58. Xu, H. et al. Metal–Organic Framework-Inspired Metal-Containing Clusters for High-Resolution Patterning. *Chem. Mater.* **30**, 4124-4133 (2018).

59. Zhang, J., Cao, K., Wang, X. S. & Cui, B. Metal-carbonyl organometallic polymers, PFpP, as resists for high-resolution positive and negative electron beam lithography. *Chem. Commun.* **51**, 17592-17595 (2015).

60. Tu, M. *et al.* Direct X-ray and electron-beam lithography of halogenated zeolitic imidazolate frameworks. *Nat. Mater.* **20**, 93-99 (2021).
